# Evolution of Vibrational Spectra in the Manganese–Silicon Clusters Mn_2_Si_*n*_, *n* = 10, 12, and 13, and Cationic [Mn_2_Si_13_]^+^

**DOI:** 10.1021/acs.jpca.1c10027

**Published:** 2022-03-03

**Authors:** Vaibhav Khanna, Roshan Singh, Pieterjan Claes, Minh Tho Nguyen, André Fielicke, Ewald Janssens, Peter Lievens, John E. McGrady

**Affiliations:** † Department of Chemistry, University of Oxford, South Parks Road, Oxford OX1 3QR, U.K.; ‡ Quantum Solid-State Physics, Department of Physics and Astronomy, KU Leuven, Celestijnenlaan 200 D, B-3001 Leuven, Belgium; ¶ Institute for Computational Science and Technology (ICST), Quang Trung Software City, Ho Chi Minh City 53151, Vietnam; § Fritz-Haber-Institut der Max-Planck-Gesellschaft, Faradayweg 4-6, 14195 Berlin, Germany; ∥ Institut für Optik und Atomare Physik, Technische Universität Berlin, Hardenbergstrasse 36, 10623, Berlin, Germany

## Abstract

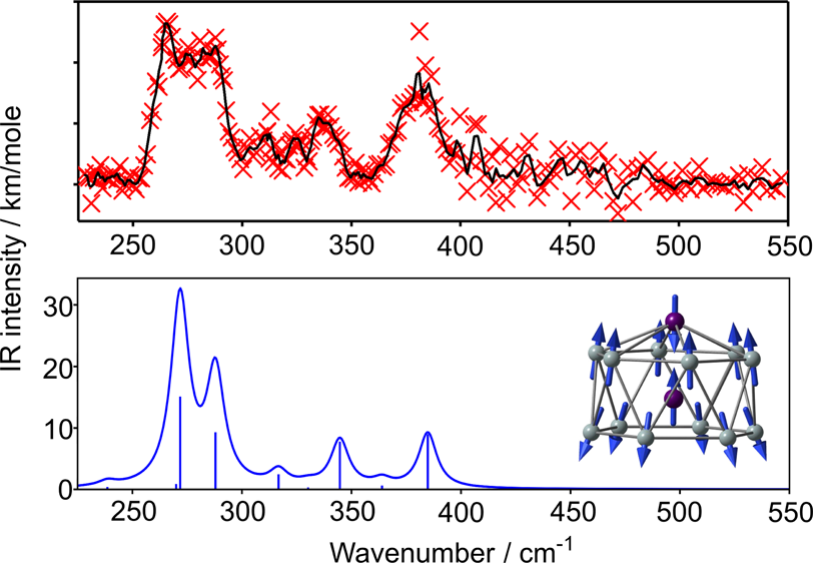

A comparison of DFT-computed and measured infrared spectra reveals the ground state structures of a series of gas-phase silicon clusters containing a common Mn_2_ unit. Mn_2_Si_12_ and [Mn_2_Si_13_]^+^ are both axially symmetric, allowing for a clean separation of the vibrational modes into parallel (a_1_) and perpendicular (e_1_) components. Information about the Mn–Mn and Mn–Si bonding can be extracted by tracing the evolution of these modes as the cluster increases in size. In [Mn_2_Si_13_]^+^, where the antiprismatic core is capped on both hexagonal faces, a relatively simple spectrum emerges that reflects a *pseudo-D*_6*d*_ geometry. In cases where the cluster is more polar, either because there is no capping atom in the lower face (Mn_2_Si_12_) or the capping atom is present but displaced off the principal axis (Mn_2_Si_13_), the spectra include additional features derived from vibrational modes that are forbidden in the parent antiprism.

## Introduction

Over the past 2 decades, the spectroscopy and electronic structure of endohedral clusters of silicon have been explored from both experimental and computational perspectives, the ultimate goal being to understand how the electronic properties of the metal impact on the cluster and *vice versa*.^1−3^ Beyond the intrinsic interest in the nature of the chemical bonds in these clusters, they can be viewed as minimal models for transition metals impurities in bulk silicon, an issue of considerable significance in the semiconductor industry. From the outset, this family of clusters has challenged conventional models of chemical bonding, and the emergence of Density Functional Theory (DFT) as a viable means of exploring potential energy surfaces has provided a platform for many studies that seek to link structure and spectroscopy to composition. There is, however, still no clear consensus on what the “best” functional is for clusters of this type, and the choice is often motivated by the authors’ previous success in related studies. The influence of Hartree–Fock exchange (present in hybrid functionals such as the popular B3LYP) on the computed energies of different spin states is well documented: larger proportions of Hartree–Fock exchange tend to favor unpaired spin density and hence higher multiplicities.^4^ Less well documented, but of particular relevance here, is the fact that even when the multiplicity is fixed, functional choice can have a substantial impact on the relative energies of different local minima, particularly in cases where the nature of the chemical bonding differs qualitatively between them. In the family of M@Si_14_ clusters (where M is the encapsulated metal dopant), for example, the BLYP functional (and its hybrid, B3LYP) tends to favor “open” structures with three-connected vertices while the PBE functional (and its hybrid, PBE0), in contrast, stabilizes deltahedral structures with more highly connected vertices.^5^ These structural differences mark a transition between localized σ/π bonding (similar to that in the carbon-based fullerenes) in the “open” structures to multicenter delocalized bonding typical of the heavier group 14 elements in the deltahedral alternatives. The balance between these two paradigms appears to be particularly delicate in silicon clusters, leading to extreme sensitivity of the relative energies of the different isomers to the choice of functional.^1−3,5−13^ Multiconfigurational self-consistent field (MC-SCF) techniques are increasingly being used as an alternative to DFT in studies of these clusters, but the balanced treatment of static and dynamic correlation required to compute meaningful energies remains a challenge.^14−22^

Given the inherent difficulties in calculating relative energies, an alternative strategy is to compare computed spectroscopic fingerprints for various candidate structures to a measured spectrum. This approach has been used with some success in the context of vibrational,^23^ photoelectron,^24−32^ and X-ray absorption spectroscopies,^33^ and it underpins much of our current understanding of these clusters. In this contribution, we use DFT as a tool to interrogate the vibrational signatures of a series of mass-selected manganese-doped silicon clusters, Mn_2_Si_10_, Mn_2_Si_12_, and Mn_2_Si_13_, along with cationic [Mn_2_Si_13_]^+^. The strong electron–electron repulsions within the 3d orbitals of the first-row transition metals are a particular challenge to DFT, and these are compounded in this case by the presence of two Mn atoms and hence the possibility of metal–metal bonding. As a result, studies of this general class of clusters have only begun to emerge in the past few years.^34−45^ Through careful comparison between experiment and theory across the Mn_2_Si_*x*_ series, we can identify the most plausible structural candidates that are consistent with the available data. Moreover, by identifying common symmetry elements, we can trace the evolution of vibrational modes through the series and connect these observations to the underlying patterns of electronic structure.

## Methodology

### Experimental Techniques

All spectroscopic measurements are performed in a molecular beam setup^46^ coupled to a beamline of the Free Electron Laser for Infrared eXperiments (FELIX) user facility.^47^ The clusters are produced in a dual-target dual-laser vaporization cluster source by pulsed ablation of manganese and silicon plate targets.^48^ Cluster–xenon complexes are formed by condensation of the vaporized material in a short pulse of He gas containing a fraction (2.5%) of isotopically enriched ^129^Xe and cooled in a thermalization channel attached to the source (115 K). Resonant absorption of IR light heats the cluster–xenon complexes through internal vibrational redistribution, which may result in dissociation of the complex. Infrared multiple photon dissociation (IR-MPD) spectra are constructed by recording the intensities of the ionic complexes as a function of the FELIX frequency in the 230–560 cm^–1^ range using a time-of-flight mass spectrometer. Neutral clusters are post-ionized, after excitation by the infrared laser and before extraction into the mass spectrometer, using a weakly focused beam of 7.87 eV photons from an F_2_ excimer laser.

### Computational Techniques

All DFT calculations were performed using the Amsterdam Density Functional (ADF) package, version 2020.103.^49^ Slater-type basis sets of triple-ζ and two polarization functions (TZ2P) were used on all atoms.^50^ The number of fit functions were increased by adding the subkey “FitType QZ4P” of the key BASIS, and a fine grid was used for numerical integration (keyword “BECKEGRID Quality good”). In previous studies on transition metal-doped silicon clusters,^14,51^ some of us have shown that the hybrid B3P86 functional gives reasonable treatment of spin states, relative energies and spectroscopic parameters. In the present work on doubly Mn-doped Si clusters, we have carried out extensive preliminary computations using both B3P86 and Perdew–Becke–Ernzerhof (PBE)^52^ functionals. B3P86 was implemented as a LibXC functional with ADF (LibXC is a library of approximate exchange-correlation functionals). After careful calibration (documented in the text), we select the PBE functional which is used in all calculations, unless stated otherwise. All calculations were performed using spin-unrestricted DFT. The initial structures were obtained in two ways: by carrying out literature surveys for reported structures of similar systems (doubly doped silicon clusters) or obtained by adding a second metal atom to previously outlined structures for singly doped silicon clusters. The computed infrared spectra were generated with a Lorentzian line shape of 5 cm^–1^ full width at half-maximum. The calculated frequency values were not scaled.

## Results and Discussion

### IR-MPD Spectroscopy of Mn_2_Si_10_, Mn_2_Si_12_, Mn_2_Si_13_, and [Mn_2_Si_13_]^+^

**Figure 1:**
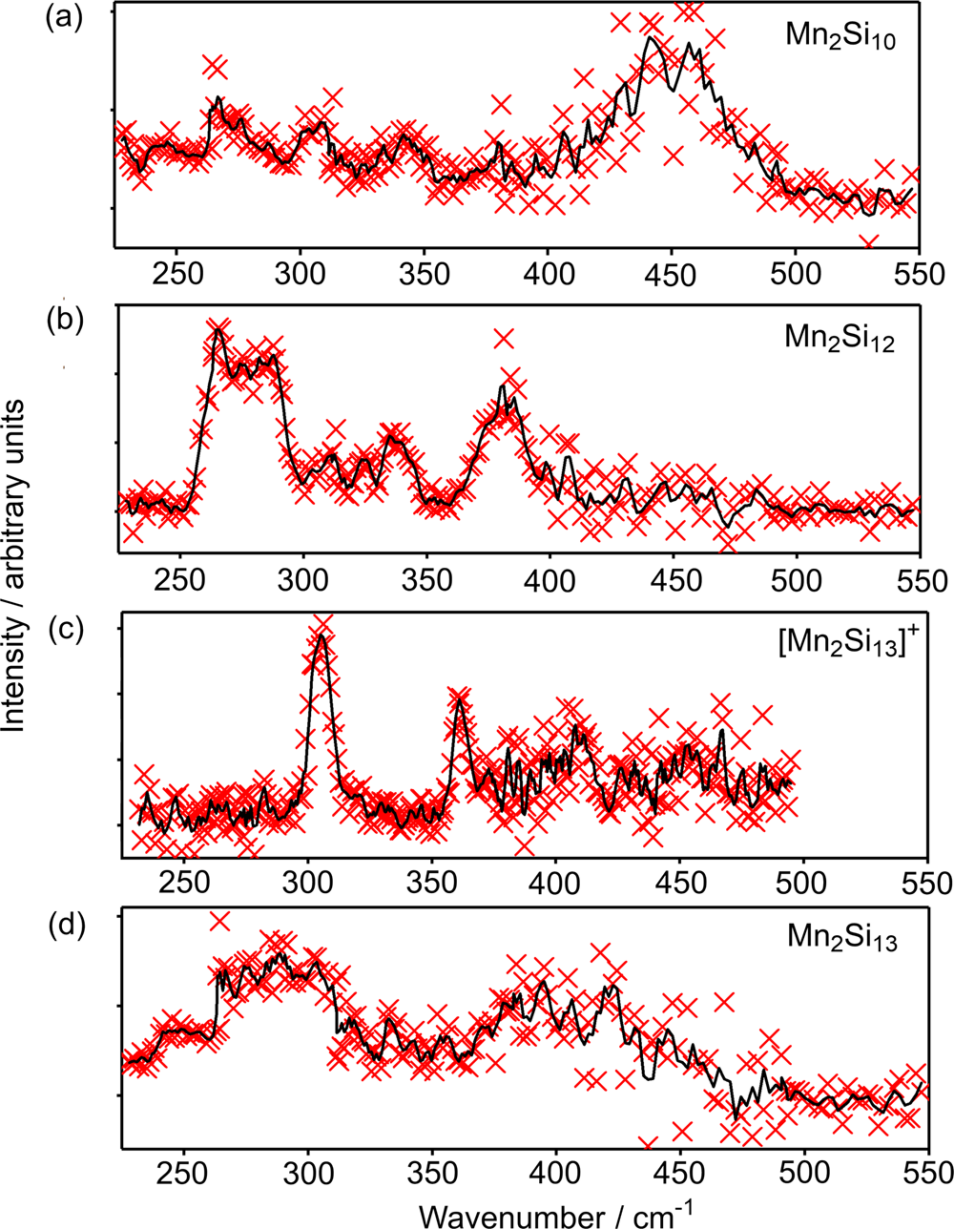
IR-MPD spectra of (a) Mn_2_Si_10_, (b) Mn_2_Si_12_, (c) [Mn_2_Si_13_]^+^, and (d) Mn_2_Si_13_. The spectra were measured by monitoring Xe loss from the corresponding Xe-tagged complexes. The experimental data points (red crosses) are overlaid with a three-point running average (black line).

IR-MPD spectra of the neutral cluster Mn_2_Si_*x*_, *x* = 10, 12, and 13, and cationic [Mn_2_Si_13_]^+^ are collected in Figure 1. The spectra of Mn_2_Si_12_ and [Mn_2_Si_13_]^+^ appear related in so much as both have their most intense feature in the window between 250 and 320 cm^–1^ and less intense bands between 325 and 400 cm^–1^. The spectrum of Mn_2_Si_13_ also features intense absorptions in the 250–300 and 375–425 cm^–1^ windows, but the bands are much less distinct. The striking similarities between the spectra suggest that the clusters may share common structural features that determine at least the gross features of the vibrational manifold. The spectrum of Mn_2_Si_12_ is also conspicuously similar to that reported previously for [Co_2_Si_12_]^+^,^53^ although the relative intensities of the bands in the low-frequency region (below 300 cm^–1^) are somewhat lower for the cobalt analogue. The spectrum of Mn_2_Si_10_ stands out as being quite different from any of the others: its most intense band is a broad feature centered at ∼450 cm^–1^, with less intense peaks at 260 and 310 cm^–1^. Again, these features are mirrored in the published spectrum of [Co_2_Si_10_]^+^, where a pronounced double peak is centered on ∼430 cm^–1^.^53^ This data set is clearly rich in information, and in the following sections, we use DFT to explore the potential energy surface and vibrational properties of these clusters with the aim of establishing the extent to which shared structural characteristics lead to similarities and differences in the measured IR-MPD spectra.

### Density Functional Theory

#### Ground-State Structure and Vibrational Spectrum of Mn_2_Si_12_

**Figure 2:**
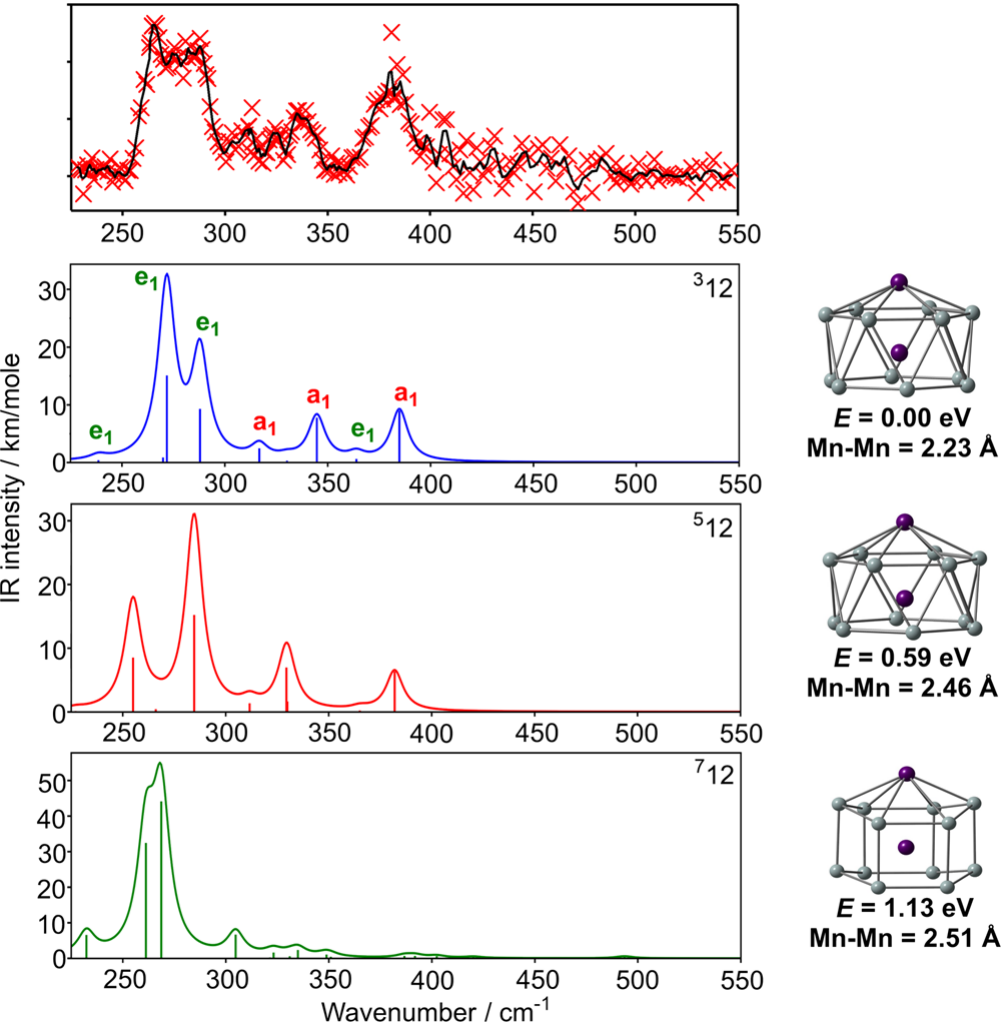
IR-MPD spectrum of the Mn_2_Si_12_·Xe complex (intensity in arbitrary units), optimized structures and computed IR spectra of the ^**3**^**12**, ^**5**^**12**, and ^**7**^**12** states of Mn_2_Si_12_, all calculated with the PBE functional.

**Figure 3:**
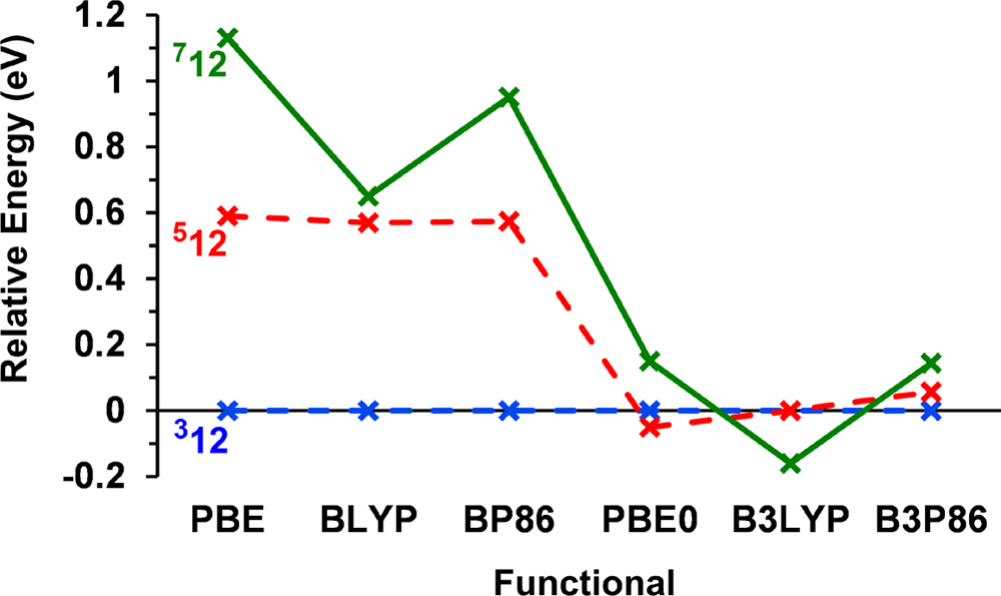
Functional dependence of the relative energies of the low-lying states (^**3**^**12**, ^**5**^**12**, ^**7**^**12**) of Mn_2_Si_12_. The ^**3**^**12** state (blue) is chosen as the point of reference for all functionals.

Our survey of the potential energy surface of Mn_2_Si_12_ (using the PBE functional) reveals two low-lying local minima with hexagonal antiprismatic geometries, one a triplet identified as ^**3**^**12** and the other a quintet, ^**5**^**12** (Figure 2). We have also identified a hexagonal prismatic septet (^**7**^**12**). All three isomers have one endohedral Mn and a second one capping a hexagonal face, with either exact or approximate *C*_6*v*_ point symmetry. The most stable of these is ^**3**^**12**, with the quintet and septet lying 0.59 and 1.13 eV higher in energy, respectively. The same hexagonal antiprismatic structure was identified as the equilibrium structure of the 59/60-electron clusters [Mo_2_Si_12_] and .^26,54^ Taken at face value, these data appear to be quite definitive in identifying the ^**3**^**12** isomer as the one observed in the experiment, but we have emphasized above the extreme sensitivity of computed total energies to functional choice. Our choice of the gradient-corrected PBE functional was based on its common usage in group-14 cluster chemistry,^3,5,39^ but there is a substantial body of work in the field that employs hybrid functionals such as B3LYP or B3P86 rather than gradient-corrected alternatives.^8,23,46,51,55^ To try to unravel the impact of different choices of functional, we have recomputed the energies of the low-lying states of Mn_2_Si_12_ using the gradient-corrected functionals BLYP and BP86 and also the hybrids, PBE0, B3LYP, and B3P86. In this way, we can separate the influence of the exchange/correlation functional from the effects of Hartree–Fock exchange. The relative energies of the three states, shown in Figure 3, reveal a complex picture, where the identity of the ground state is indeed highly dependent on functional choice. While PBE, BLYP, BP86, and B3P86 concur in identifying the ^**3**^**12** isomer as the ground state, PBE0 favors the quintet, ^**5**^**12**, and B3LYP predicts the hexagonal prismatic septet, ^**7**^**12**. The influence of Hartree–Fock exchange is immediately apparent: it stabilizes the quintet relative to the triplet and the septet relative to the quintet, the result being that the relative energies of the three states are very close for all three hybrids. A second, more subtle, feature is that the LYP correlation functional (in BLYP and B3LYP) stabilizes the ^**7**^**12** isomer by ∼0.5 eV relative to the other two, with the result that this isomer emerges as the global minimum only for B3LYP. This is precisely the trend identified previously in the family of M@Si_14_ clusters, where the BLYP functional (and its hybrid) tends to favor “open” structures with three-connected vertices (in this case the hexagonal prism) over those with more highly connected vertices (in this case the hexagonal antiprism). The trends identified in Figure 3 are also apparent in the work of Khanna and co-workers on Fe_2_Si_12_^39^ and Liang et al. on [Cr_2_Ge_12_]^−^.^31^ In the first of these, using the PBE functional, the hexagonal antiprismatic architecture was clearly the most stable whereas in the second, performed with B3P86, the hexagonal prism was identified as a very low-lying transition state that could facilitate rapid rearrangement. Our conclusion here is simply that the calculated total energies using any single functional are a poor criterion on which to base an assignment of the ground-state structure.

The computed IR spectrum of the ^**3**^**12** isomer (the blue spectrum in Figure 2) appears to provide a good match to the experimental data. Prominent bands at 272 and 288 cm^–1^ (both with e_1_ symmetry) map on to the broad experimental feature between 260 and 290 cm^–1^, while the a_1_-symmetric modes at 345 and 385 cm^–1^ are consistent with features at ∼340 cm^–1^ and ∼380 cm^–1^, respectively. We show later that the 345 cm^–1^ mode has significant Mn–Mn stretching character and is a direct signature of the presence of the Mn–Mn bond. Although less prominent, an a_1_-symmetric vibration at 317 cm^–1^ also corresponds to a less intense experimental feature around 320 cm^–1^. The ^**5**^**12** isomer shows very similar features, although the splitting of the two low-frequency peaks is rather more pronounced. The ^**7**^**12** alternative also has prominent bands in the 250–300 cm^–1^ region, but the spectrum is devoid of intense features above 350 cm^–1^, and it does not, therefore, offer an obvious assignment for the peak observed around 380 cm^–1^ in the experimental data. On this basis, it seems that the ^**3**^**12** isomer is the most plausible candidate for the experimentally observed cluster. As a corollary to this observation, the PBE functional (along with BP86 and BLYP) appears to identify the correct ground state isomer while the B3LYP functional does not.

**Figure 4:**
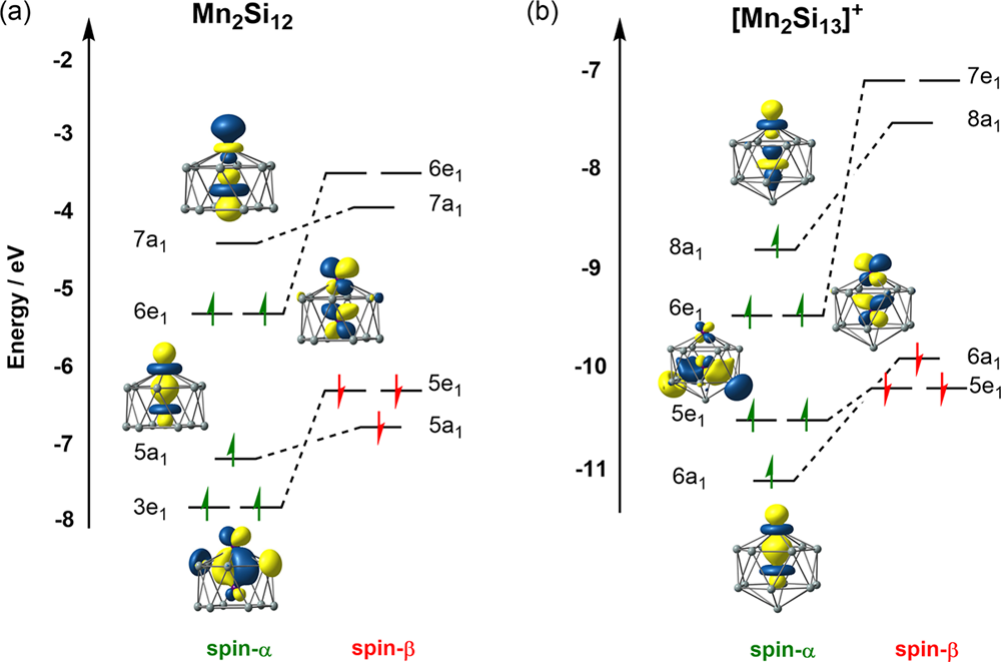
Frontier Kohn–Sham orbitals for (a) the ^**3**^**12** state of Mn_2_Si_12_ and (b) the ^**4**^**13**^**+**^(a) state of [Mn_2_Si_13_]^+^.

The identification of a triplet ground state in Mn_2_Si_12_ indicates that the majority of the intrinsic magnetic moments of the component Mn atoms are quenched. The role of the Si_12_ cage in quenching the moment of the endohedral metal has been explored in detail by Khanna and co-workers in the context of CrSi_12_^10,56^ and also by us in the context of isoelectronic [MnSi_12_]^+^:^15^ in short, there is very substantial covalence that delocalizes the electron density onto the cage, favoring spin pairing. As a result, any residual spin moment is localized strongly on the external Mn ion, which is only partially coordinated by one Si_6_ hexagon. A spin density plot for Mn_2_Si_12_ is shown in the Supporting Information, Figure S1, and the projected Mulliken spin densities (PBE functional) are +2.83 and −0.22 on the external and endohedral Mn centers, respectively, with a further 0.61 spin-β electrons localized on the Si_12_ cage. The Mn–Mn separation of 2.23 Å in the ^**3**^**12** ground state is much shorter than those for typical Mn–Mn single bonds (2.895 Å in Mn_2_(CO)_10_, for example^57^) and, while bond-length-bond-order correlations are notoriously difficult to establish with certainty when bridging ligands (such as Si_12_, here) are present, such a short bond is certainly indicative of strong Mn–Mn bonding. The frontier Kohn–Sham orbitals for the triplet ground state in Figure 4(a) include a doubly occupied Mn–Mn σ orbital, 5a_1_, and also a doubly occupied degenerate Mn–Mn π* orbital, 6e_1_α, and hence a formal Mn=Mn double bond, qualitatively similar to that in O_2_. Much like the calculated energies of the various states, these computed Mulliken spin densities (and also the values of ⟨*S*^2^⟩) are extremely sensitive to functional choice, and the corresponding values with the hybrid PBE0 (+4.24 and −2.73 on external and endohedral Mn, respectively) indicate a much more extreme degree of polarization. This striking functional dependence indicates substantial static correlation that we will explore in a forthcoming study using multi-configurational SCF techniques.

#### Ground-State Structure and Vibrational Spectrum of [Mn_2_Si_13_]^+^

**Figure 5:**
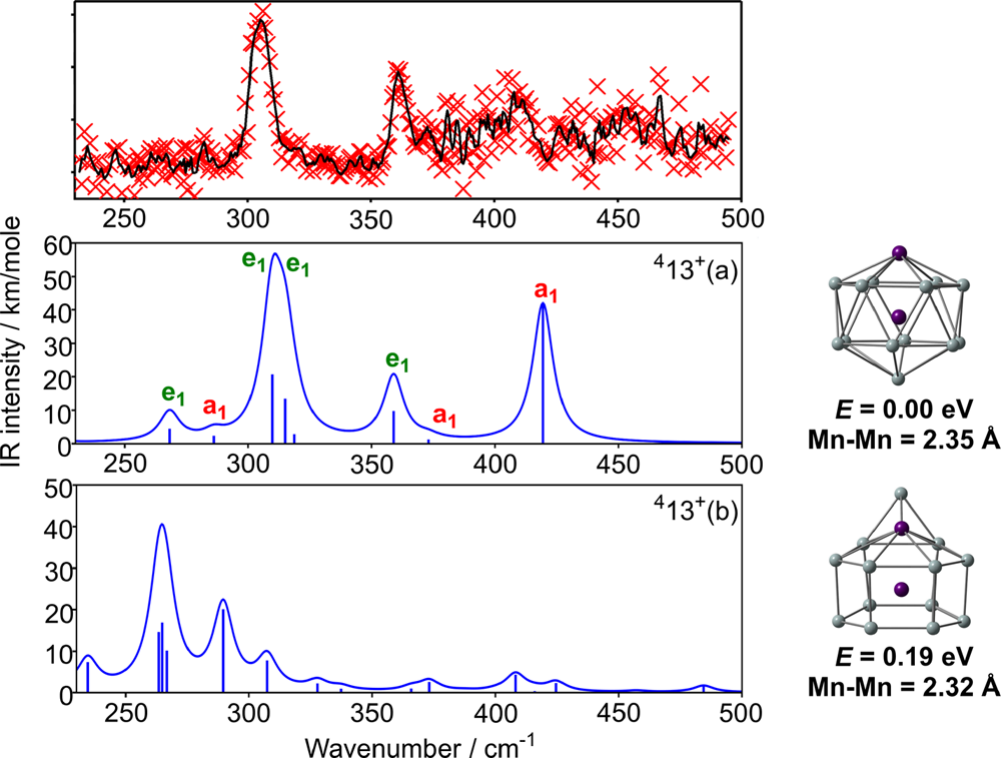
IR-MPD spectrum of [Mn_2_Si_13_]^+^·Xe complex (intensity in arbitrary units), optimized structures and computed IR spectra of the low-lying ^**4**^**13**^**+**^(a) and ^**4**^**13**^**+**^(b) isomers of [Mn_2_Si_13_]^+^ (PBE functional).

The optimized geometries of two low-lying states of [Mn_2_Si_13_]^+^ are collected in Figure 5, along with their computed vibrational fingerprints. The energies reported in the figure relate to the PBE functional, which we adopt, on the basis that it identified the equilibrium structure of Mn_2_Si_12_ correctly. Our survey of the potential energy surface identifies as the global minimum a *C*_6*v*_-symmetric isomer, ^**4**^**13**^**+**^(a), derived, at least conceptually, from the Mn_2_Si_12_ antiprism by capping the remaining hexagonal face with a Si^+^ ion. The axial symmetry again allows for a clean separation of the vibrational peaks into parallel (a_1_) and perpendicular (e_1_) modes, and the two prominent peaks in the experimental spectrum are readily assigned to the computed modes at 310/315 and 359 cm^–1^, all of which have e_1_ symmetry. The alternative ’prism-like’ isomer, ^**4**^**13**^**+**^(b), is 0.19 eV higher in energy with the PBE functional, and the computed spectrum of the ^**4**^**13**^**+**^(b) isomer offers a significantly worse match to experiment. For this reason, we assign the ground state as ^**4**^**13**^**+**^(a). It is worth noting, however, that the ^**4**^**13**^**+**^(b) is computed to be more stable than ^**4**^**13**^**+**^(a) for all other functionals tested (Figure S2), again underlining the pitfalls of relying solely on computed relative energies to identify structure. The spin density in the ^**4**^**13**^**+**^(a) ground state is again localized strongly on the external Mn ion (+3.41) while the moment at the endohedral metal remains largely quenched (+0.67). The presence of the additional Si atom on the principal axis serves to weaken the Mn–Mn bond compared to Mn_2_Si_12_, and the Mn–Mn σ* orbital shown in Figure 4b is now singly occupied. As a result, the Mn–Mn bond length is 2.35 Å, 0.12 Å longer than in Mn_2_Si_12_. Unlike the Mn_2_Si_12_ case, there is no obvious signature of the Mn–Mn bonding in the vibrational spectrum: all modes with significant Mn–Mn stretching character are found at very low frequencies and with low intensities, and in fact the only a_1_-symmetric mode with substantial intensity (at 419 cm^–1^) has dominant Mn–Si, rather than Mn–Mn, character. The experimental spectrum above 400 cm^–1^ does not show well-defined peaks, but there is evidence for an increase in intensity at ∼410 cm^–1^ that we assign to this mode.

#### Symmetry Analysis and Comparison of Mn_2_Si_12_ and [Mn_2_Si_13_]^+^

The fact that the computed ground states of Mn_2_Si_12_ and [Mn_2_Si_13_]^+^ share a hexagonal antiprismatic core with a common 6-fold rotation axis allows for a clean separation between modes involving motion along the principal axis and perpendicular to it. The uncapped hexagonal antiprism, [MnSi_12_]^+^, therefore represents a natural reference point for the following discussion. The [MnSi_12_]^+^ cation has been the subject of a number of studies in its own right, and the ground state is in fact a hexagonal prism rather than the antiprism that we consider here, which is located 0.46 eV higher in energy (PBE). The [MnSi_12_]^+^ anti-prism has open-shell singlet and triplet states that lie within 0.01 eV: to avoid complications due to spin contamination in the open-shell singlet, we use the triplet here. The fact that the hexagonal anti-prism is not the ground state of [MnSi_12_]^+^ is not critical here: its role is simply to act as a reference point for the discussion of the capped analogues that are known.

**Figure 6:**
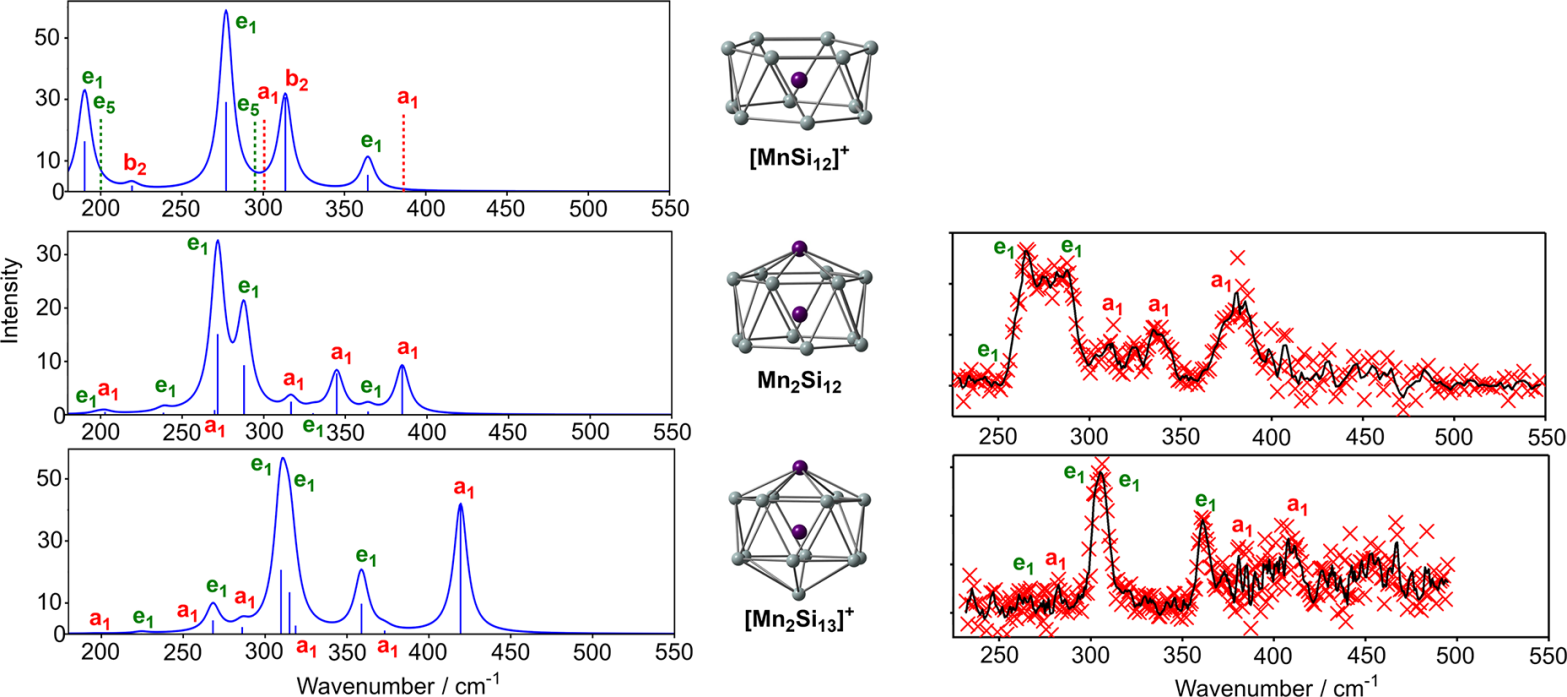
Evolution of the vibrational spectra of [MnSi_12_]^+^, Mn_2_Si_12_, and [Mn_2_Si_13_]^+^.

**Figure 7:**
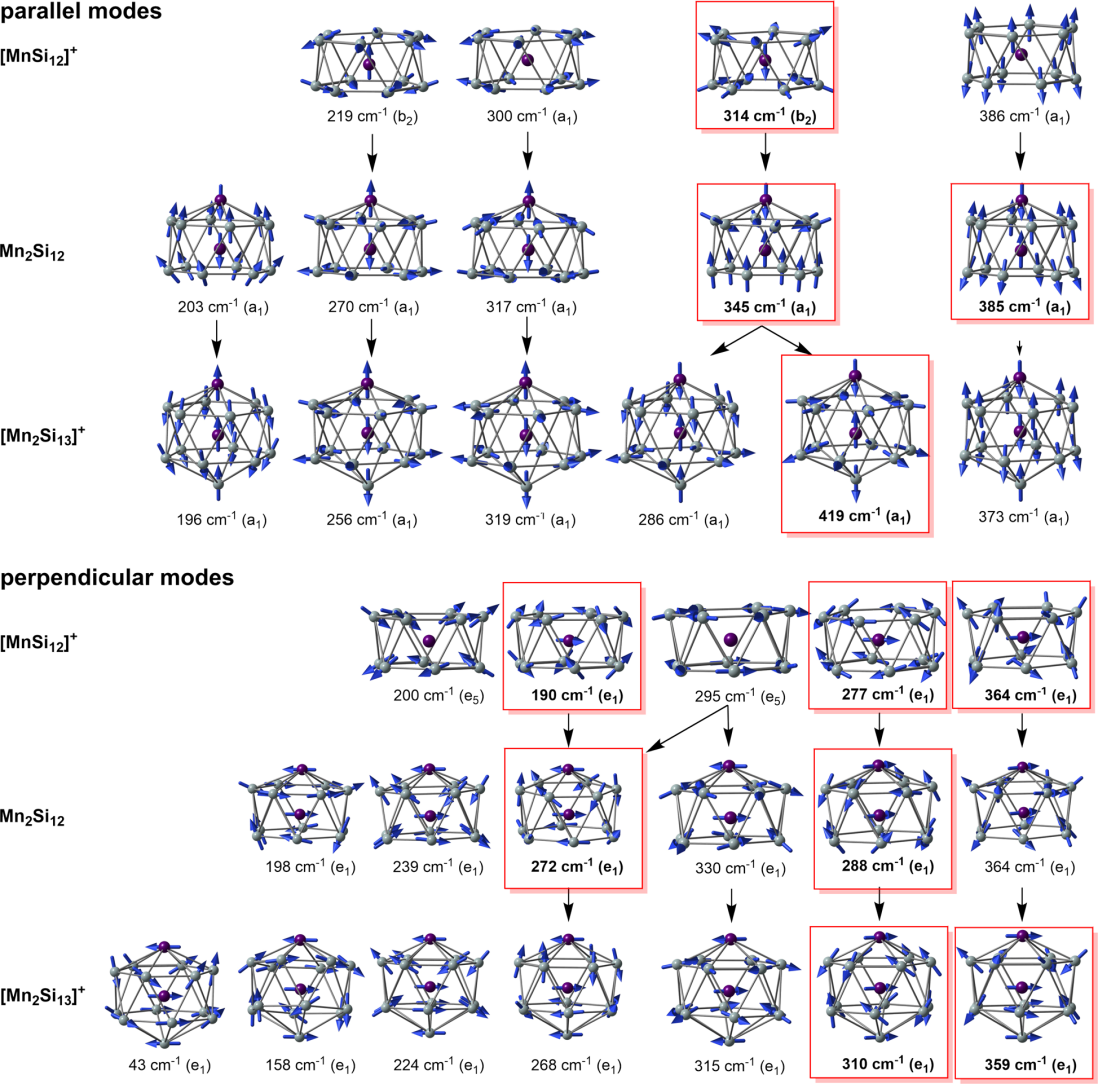
Evolution of the parallel and perpendicular vibrational modes in the series [MnSi_12_]^+^, Mn_2_Si_12_, and [Mn_2_Si_13_]^+^. Modes that feature prominently in the spectra are enclosed in red boxes.

The spectra (experimental and computed) of Mn_2_Si_12_ and [Mn_2_Si_13_]^+^ are compared in Figure 6, alongside the computed spectrum of the antiprismatic isomer of [MnSi_12_]^+^. Atomic displacements for the significant vibrational modes are shown in Figure 7, where they are separated into parallel (a_1_) and perpendicular (e_1_) subsets. Modes that feature prominently in the experimental spectrum are enclosed in red boxes. The spectrum of the [MnSi_12_]^+^ reference shows many of the features noted above for Mn_2_Si_12_ and [Mn_2_Si_13_]^+^, most notably an intense e_1_-symmetric vibration at 277 cm^–1^ that involves the motion of the endohedral Mn in the *xy* plane. There are two further modes of e_1_ symmetry that carry significant intensity, at 190 and 364 cm^–1^, both involving canting of the two Si_6_ rings. Among the parallel set, an intense b_2_-symmetric mode at 314 cm^–1^ involves motion of the Mn atom along the principal axis, coupled to an out-of-phase contraction/expansion of the two Si_6_ rings. Although not allowed in the infrared spectrum of *D*_6*d*_-symmetric [MnSi_12_]^+^, modes of a_1_ and e_5_ symmetry are also shown in the figures because they correlate with a_1_ and e_1_ modes in *C*_6*v*_ symmetry, and so become allowed when capping atoms are introduced in Mn_2_Si_12_ and [Mn_2_Si_13_]^+^. Among these, the two a_1_-symmetric modes are breathing modes of the Si_12_ cage, either in the *xy* plane (300 cm^–1^) or along the principal axis (386 cm^–1^). The e_5_ modes involve either canting of the rings (200 cm^–1^) or deformation of the individual Si_6_ rings (295 cm^–1^).

The intense 277 cm^–1^ perpendicular mode in [MnSi_12_]^+^ can be tracked through the 288 cm^–1^ mode in Mn_2_Si_12_ and then to the 310 cm^–1^ mode in [Mn_2_Si_13_]^+^, all of which are dominated by motion of the Mn atom in the *xy* plane. Similarly, the characteristic canting of the rings in the 364 cm^–1^ mode in [MnSi_12_]^+^ is also found in the 364 cm^–1^ mode of Mn_2_Si_12_ and also the 359 cm^–1^ mode of [Mn_2_Si_13_]^+^. The most striking feature of the spectrum of Mn_2_Si_12_ is the appearance of an additional e_1_-symmetric mode with high intensity, at 272 cm^–1^, which is responsible for the striking broadening in the experimental spectrum in the 250–300 cm^–1^ window. The displacements in this mode show that it is derived from a linear combination of the e_1_ and *e*_5_-symmetric modes of [MnSi_12_]^+^ at 190 and 295 cm^–1^, respectively, both of which transform as e_1_ in *C*_6*v*_ symmetry. The “e_5_” character is apparent in the lower Si_6_ ring while the “e_1_” character is localized in the upper ring. The significant intensity of this mode (the most intense peak in the computed spectrum) is a direct consequence of the presence of the capping Mn atom: without it, the intensity drops to zero. We can follow the same displacements through to the 268 cm^–1^ mode of [Mn_2_Si_13_]^+^, which has much lower intensity and is barely discernible above the baseline in the experiment. The reduction in intensity compared to Mn_2_Si_12_ signals a return to a more symmetric, “*pseudo*-*D*_6*d*_” environment for the cage, where both Si_6_ faces are capped: the intensity would tend to zero in the limit that the Mn and Si caps were electronically identical.

A similar pattern of behavior can be identified in the parallel modes. The intense b_2_-symmetric peak at 314 cm^–1^ in [MnSi_12_]^+^ can be tracked into the 345 cm^–1^ mode of Mn_2_Si_12_, and from there to the antisymmetric Si–Mn–Mn stretch at 419 cm^–1^ in [Mn_2_Si_13_]^+^. The corresponding symmetric stretch at 286 cm^–1^ is much less intense, but a small feature in this region just discernible above the baseline in the experimental spectrum. The second prominent band of a_1_ symmetry in the spectrum of Mn_2_Si_12_, at 385 cm^–1^, is closely related to the axial symmetric breathing of [MnSi_12_]^+^ at 386 cm^–1^ which was strictly forbidden in *D*_6*d*_ symmetry. This mode therefore owes its intensity entirely to the polar environment created by the capping Mn atom, and it vanishes again when the counterbalancing Si cap is introduced in [Mn_2_Si_13_]^+^ (373 cm^–1^). The forbidden a_1_-symmetric equatorial breathing mode of [MnSi_12_]^+^ at 300 cm^–1^ also “lights up” in the presence of the capping Mn atom of Mn_2_Si_12_, in the 317 cm^–1^ mode that appears as a weak feature in the experimental spectrum. The corresponding 319 cm^–1^ mode in [Mn_2_Si_13_]^+^ is reduced in intensity, again signaling a return to a *pseudo*-*D*_6*d*_ symmetric environment.

When considered against the reference point of the rigorously *D*_6*d*_-symmetric [MnSi_12_]^+^ antiprism, it becomes clear that we can understand the spectra of Mn_2_Si_12_ and [Mn_2_Si_13_]^+^ in terms of the extent to which the antiprismatic MnSi_12_ core deviates from the high-symmetry *D*_6*d*_ limit. The intense, allowed, bands involving motion of the endohedral Mn atom in the *xy* plane are apparent in both spectra (288 and 364 cm^–1^ in Mn_2_Si_12_; 310 and 359 cm^–1^ in [Mn_2_Si_13_]^+^) while the parallel motion of the endohedral Mn atom in [MnSi_12_]^+^ (314 cm^–1^) shifts into an Mn–Mn stretch at 345 cm^–1^ in Mn_2_Si_12_ and an antisymmetric Si–Mn–Mn stretch at 419 cm^–1^ in [Mn_2_Si_13_]^+^. The spectrum of Mn_2_Si_12_ contains additional features because the polar environment allows bands that are formally forbidden in *D*_6*d*_ symmetry to acquire significant intensity. Thus, we see new features at 272 cm^–1^ (e_1_), 317 and 385 cm^–1^ (both a_1_) that can be traced to modes that are formally forbidden at the high-symmetry limit. All three of these extra bands are discernible in the experimental spectrum. The fact that these additional features disappear in the spectrum of [Mn_2_Si_13_]^+^ reflects the rather similar electronic effects of the capping Mn and Si atoms, which restore approximate *D*_6*d*_ symmetry to the antiprismatic MnSi_12_ core. The more complex spectrum of Mn_2_Si_12_ compared to that of [Mn_2_Si_13_]^+^ is, therefore, a clear indication of the more polar, less symmetric, structure of the former.

#### Vibrational Spectrum of Mn_2_Si_13_

**Figure 8:**
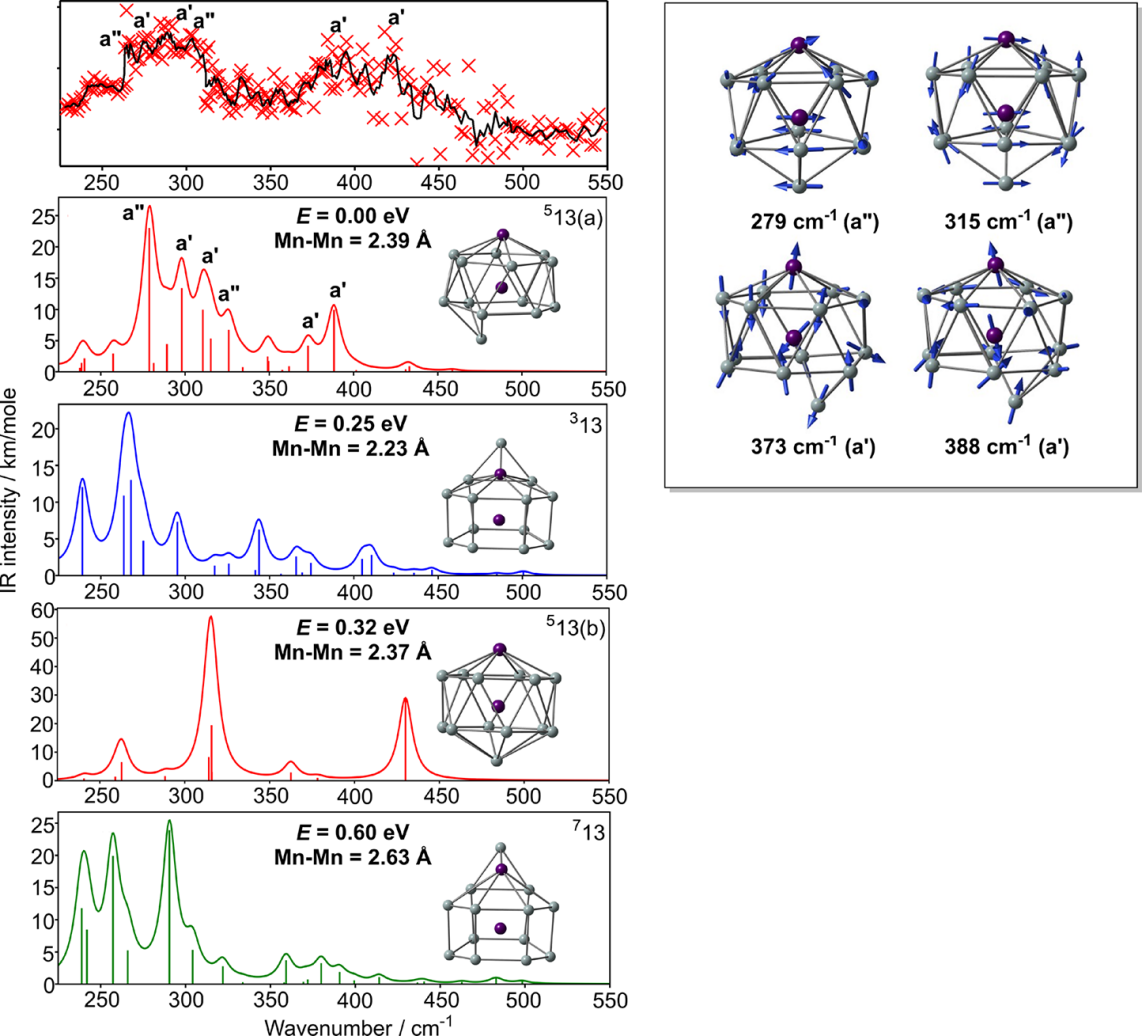
IR-MPD spectrum of the Mn_2_Si_13_·Xe complex (intensity in arbitrary units), optimized structures and computed IR spectra of the low-lying isomers of Mn_2_Si_13_ (PBE functional). Vibrational modes corresponding to intense peaks in the computed spectrum of ^**5**^**13(a)** are also shown. Note that ^**5**^**13(b)** is a second order saddle point, not a minimum.

The IR-MPD spectrum of Mn_2_Si_13_ has a lower signal-to-noise ratio than those reported above, but nevertheless we can identify similar features, notably a region of high intensity in the 275–300 cm^–1^ region and another between 375 and 425 cm^–1^. The very broad peaks are immediately indicative of a lower symmetry structure, and indeed, we find that while the lowest energy structure, ^**5**^**13(a)**, has the same bicapped hexagonal antiprismatic structure as the corresponding cation, the capping Si atom has moved off the principal axis (Mn–Mn–Si = 159.5°) to give an approximately *C*_*s*_-symmetric structure (Figure 8). The *C*_6*v*_-symmetric structure (^**5**^**13(b)** in Figure 8) has multiple imaginary frequencies which stem from an orbitally degenerate ground state. The reduction in symmetry splits all of the degenerate perpendicular vibrations into distinct a′ and a″ components, and also amplifies the difference between the upper and lower Si_6_ hexagonal faces. The result is a return to a spectrum typical of Mn_2_Si_12_, where the modes involving motion of the endohedral Mn are complemented by additional features that would be forbidden in the antiprism itself. The additional (and, as in Mn_2_Si_12_, more intense) bands are exemplified by the a″ mode at 315 cm^–1^, the analogue of the 272 cm^–1^ mode in Mn_2_Si_12_, and the a′ mode at 373 cm^–1^, the analogue of the 385 cm^–1^ breathing mode in Mn_2_Si_12_. The 388 cm^–1^ mode contains a large contribution from the asymmetric Mn–Mn–Si stretch seen at 419 cm^–1^ in the cation, the lower frequency reflecting the displacement of the capping Si off the principal axis. The striking similarities between the spectra of Mn_2_Si_13_ and Mn_2_Si_12_ therefore reflect a common polar structure where the upper and lower hexagonal faces are sharply differentiated, compared to the rather simpler spectrum of [Mn_2_Si_13_]^+^ where the antiprism is more symmetrically capped.

#### Vibrational Spectrum of Mn_2_Si_10_

**Figure 9:**
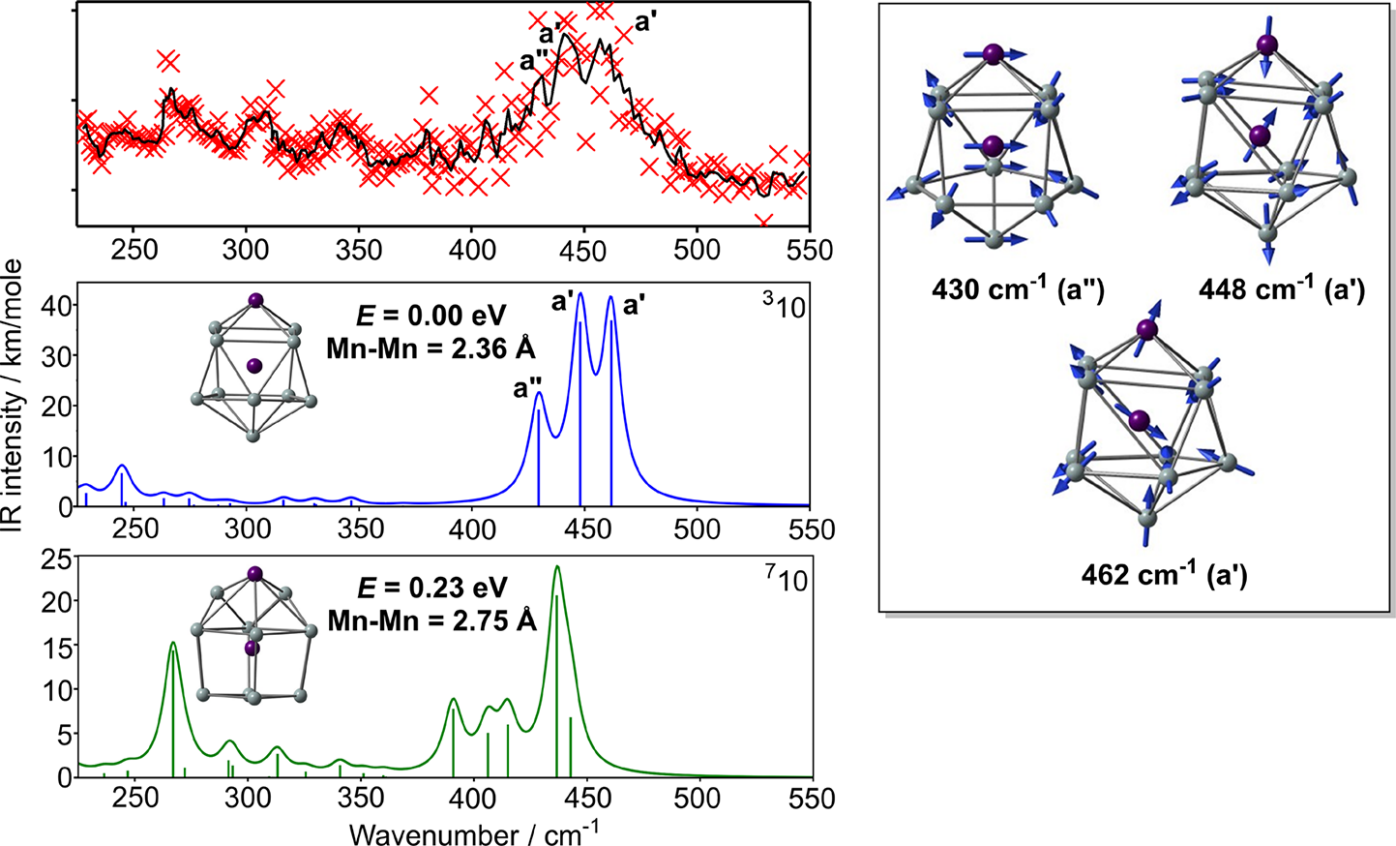
IR-MPD spectrum of the Mn_2_Si_10_·Xe complex (intensity in arbitrary units), optimized structures and computed IR spectra of the low-lying isomers of Mn_2_Si_10_. Vibrational modes corresponding to intense peaks in the computed spectrum of ^**3**^**10** are also shown (PBE functional).

Finally, we turn to the spectrum of Mn_2_Si_10_, which is strikingly different from all others in so much as the maximum intensity is found above 400 cm^–1^. The structures of two low-lying isomers of Mn_2_Si_10_, ^**3**^**10** and ^**7**^**10**, both with *C*_*s*_ symmetry, are compared in Figure 9. While the lower symmetry makes it harder to classify the structures, it is clear that the connectivity of the Si vertices in ^**3**^**10** is high (4 or 5) and in that sense the structure resembles the hexagonal antiprism of Mn_2_Si_12_ (connectivity 4) rather more than the prism (connectivity 3). Conversely, the ^**7**^**10** isomer bears closer resemblance to the prisms in the sense that the Si vertices are 2, 3, or 4 connected, and indeed both the multiplicity (7) and the Mn–Mn bond length (2.75 Å) resemble those in the ^**7**^**12** structure of Mn_2_Si_12_. With the PBE functional, the ^**3**^**10** isomer is the more stable of the two, with the ^**7**^**10** state 0.23 eV higher, but the situation is reversed for the hybrids, PBE0 and B3LYP, where the ^**7**^**10** isomer is the more stable by 0.25 and 0.42 eV, respectively (see Supporting Information, Figure S4). We see precisely the same systematic bias of the LYP correlation functional toward low-connectivity structures that was identified for all other clusters considered in this paper. Again, we conclude that the computed energies are highly functional dependent, and they are therefore an unreliable metric upon which to base any assignment of the ground state.

The comparison between the measured IR-MPD spectrum and the DFT-computed fingerprints, also shown in Figure 9, indicates that the ^**3**^**10** isomer provides a good match to the experiment, with three bands in the 425–475 cm^–1^ window, coincident with the broad absorption feature observed in this region. We also note here that the rather similar spectrum of [Co_2_Si_10_]^+^ was previously assigned to a structure almost identical with ^**3**^**10**, despite the fact that it was computed (using the hybrid B3P86 functional) to be less stable than the analogue of ^**7**^**10**.^53^ The absence of a 3-fold or higher rotational axis again splits the modes involving motion perpendicular to the principal axis into distinct a′ and a″ components, and also mixes the parallel and perpendicular modes in a′ symmetry. Nevertheless, we can identify the perpendicular motion of the Mn atom in the a″ mode at 430 cm^–1^, while the other two intense features at 448 and 462 cm^–1^ have mixed parallel and perpendicular character. The major impact of the smaller cage (Si_10_ vs Si_12_) is therefore to shift the perpendicular modes to higher frequencies by ∼150 cm^–1^ where they mix with the parallel modes that are found in a similar region in all of the spectra discussed previously.

## Summary and Conclusions

In this work we have measured the IR-MPD spectra of a series of silicon clusters containing a Mn_2_ unit, Mn_2_Si_*x*_, with *x* = 10, 12, and 13, and also the cation [Mn_2_Si_13_]^+^. By comparison of these spectra to the DFT-computed fingerprints of various candidate structures, we have been able to identify the isomers which offer the best match to experiment. In all cases, these are based on antiprismatic architectures, which are identified consistently as the most stable only by the PBE functional. The B3LYP functional, in contrast, shows a systematic bias toward alternative structures with low-connectivity vertices (prisms rather than antiprisms).

The ground-state structures of Mn_2_Si_12_ and [Mn_2_Si_13_]^+^ are both axially symmetric, which allows us to trace the evolution of the vibrational modes involving motion parallel (a_1_) and perpendicular (e_1_) to the principal axis. The isomeric *D*_6*d*_-symmetric hexagonal antiprismatic [MnSi_12_]^+^ is a useful reference point in this regard because the high symmetry leads to a relatively simple vibrational spectrum. The major peaks in the spectrum of [Mn_2_Si_13_]^+^ have very similar character (in terms of atomic displacements) to those in [MnSi_12_]^+^, reflecting the fact that the capping Mn and Si atoms on opposite hexagonal Si_6_ faces exert a rather similar electronic influence on the antiprism, giving a *pseudo*-*D*_6*d*_ environment. The vibrational spectrum of Mn_2_Si_12_, in contrast, shows a number of additional features that can be traced to modes that are forbidden in *D*_6*d*_ symmetry but acquire intensity in the polar *C*_6*v*_ environment imposed by the single capping Mn atom. In particular, the prominent peak around 380 cm^–1^ corresponds to the symmetric breathing mode of the Si_12_ cage, and is the clearest manifestation of the reduced symmetry. The neutral Mn_2_Si_13_ cluster has a similar structure to the cation, except that the capping Si atom has moved off the principal axis. The resulting loss of axial symmetry splits the degenerate modes into two, and also amplifies the difference between the upper and lower Si_6_ faces that led to a more complex spectrum in Mn_2_Si_12_. It is no coincidence, therefore, that the IR-MPD spectra of Mn_2_Si_12_ and Mn_2_Si_13_ are striking similar, both showing high-intensity features that correspond to vibrations that are forbidden in the parent antiprism. Our analysis shows that these spectra are much more than mere fingerprints, and, when fully assigned, they are rich in information about structure and also the interplay between Mn–Mn, Mn–Si, and Si–Si bonding.

## Supporting Information Available

Figure S1, the spin density plots and Mulliken projected spin densities of Mn_2_Si_12_, [Mn_2_Si_13_]^+^, Mn_2_Si_13_, and Mn_2_Si_10_ (with PBE and PBE0 functionals), and Figures S2–S4, showing the functional dependence of the relative energies of the various isomers of [Mn_2_Si_13_]^+^, Mn_2_Si_13_, and Mn_2_Si_10_, respectively (PDF)
Optimized Cartesian coordinates for all structures (ZIP)
Animated gifs of all vibrational modes described in the text (ZIP)
