# Supplementary material for: Evolution of Vibrational Spectra in the Manganese–Silicon Clusters Mn2Sin, n = 10, 12, and 13, and Cationic [Mn2Si13]+
Source: J Phys Chem A. 2022 Mar 3;126(10):1617–26. doi: 10.1021/acs.jpca.1c10027 (PMC9084549; doi:10.1021/acs.jpca.1c10027)
Supplement: Supplementary file 1 — jp1c10027_si_001.pdf [file jp1c10027_si_001.pdf]

Supporting Information

Evolution of Vibrational Spectra in the Manganese-Silicon Clusters Mn_2Si_n, n = 10, 12, 13 and cationic $[Mn_2Si_{13}]^+$

Vaibhav Khanna,† Roshan Singh,† Pieterjan Claes,‡ Minh Tho Nguyen,¶ André Fielicke,§,‖ Ewald Janssens,‡ Peter Lievens,*,‡ and John E. McGrady*,†

†Department of Chemistry, University of Oxford, South Parks Road, Oxford OX1 3QR, U.K.

‡Quantum Solid-State Physics, Department of Physics and Astronomy, KU Leuven, Celestijnenlaan 200 D, B-3001 Leuven, Belgium

¶Institute for Computational Science and Technology (ICST), Quang Trung Software City, Ho Chi Minh City, Vietnam

§Fritz-Haber-Institut der Max-Planck-Gesellschaft, Faradayweg 4-6, 14195 Berlin, Germany

‖Institut für Optik und Atomare Physik, Technische Universität Berlin, Hardenbergstr. 36, 10623, Berlin, Germany

E-mail: peter.lievens@kuleuven.be; john.mcgrady@chem.ox.ac.uk

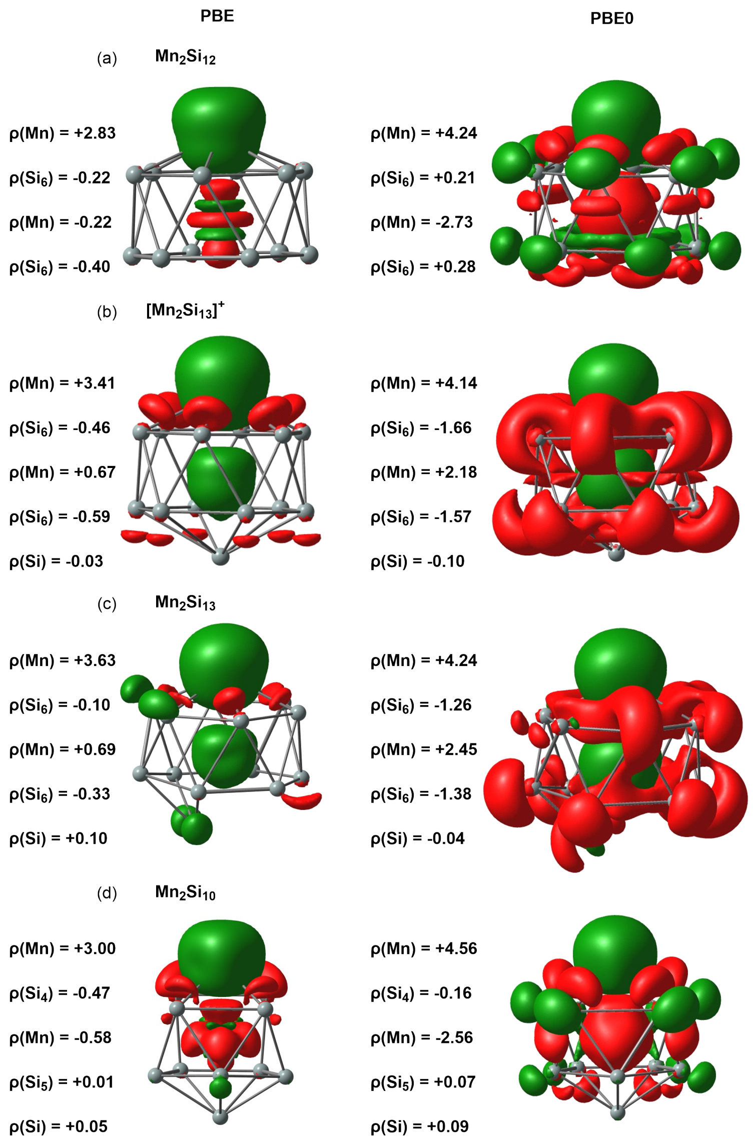

Figure S1: Spin density plots and Mulliken projected spin densities of Mn_2Si_{12}, $[Mn_2Si_{13}]^+$, Mn_2Si_{13} and Mn_2Si_{10}.

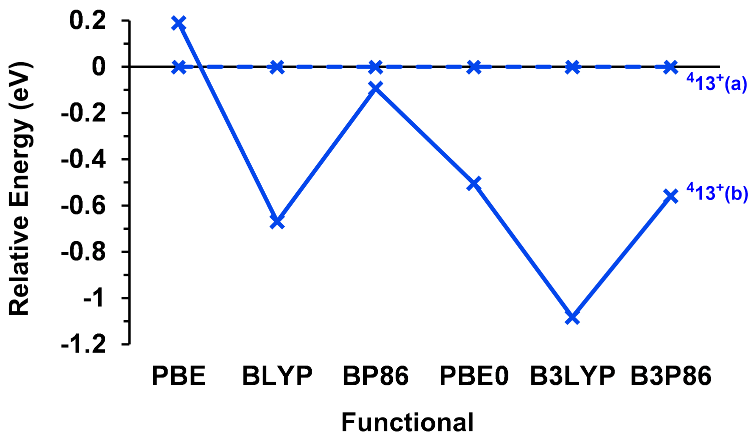

Figure S2: Variation of relative energies of different isomers of $[Mn_2Si_{13}]^+$ with different functionals.

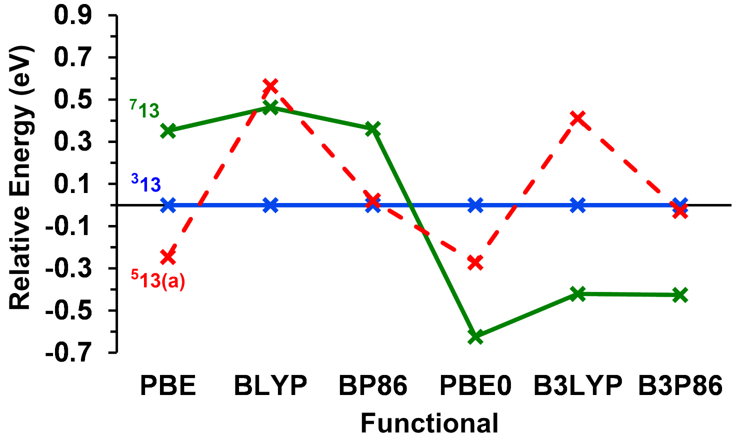

Figure S3: Variation of relative energies of different isomers of Mn_2Si_{13} with different functionals.

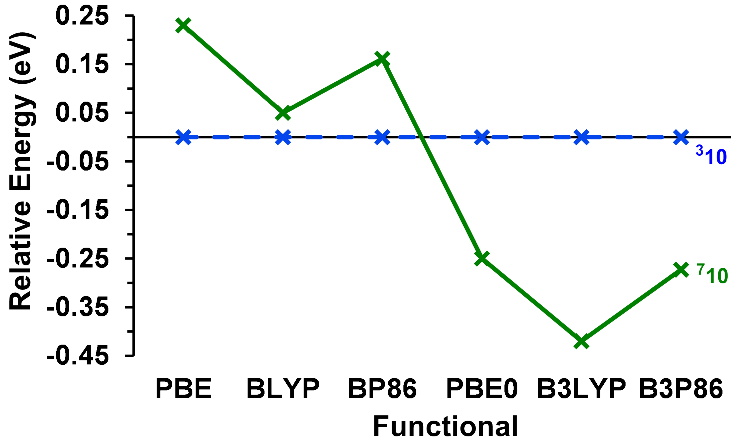

Figure S4: Variation of relative energies of different isomers of Mn_2Si_{10} with different functionals.
