# Supplementary figures and images for: Evolution of Vibrational Spectra in the Manganese–Silicon Clusters Mn2Sin, n = 10, 12, and 13, and Cationic [Mn2Si13]+
Source: J Phys Chem A. 2022 Mar 3;126(10):1617–26. doi: 10.1021/acs.jpca.1c10027 (PMC9084549; doi:10.1021/acs.jpca.1c10027)

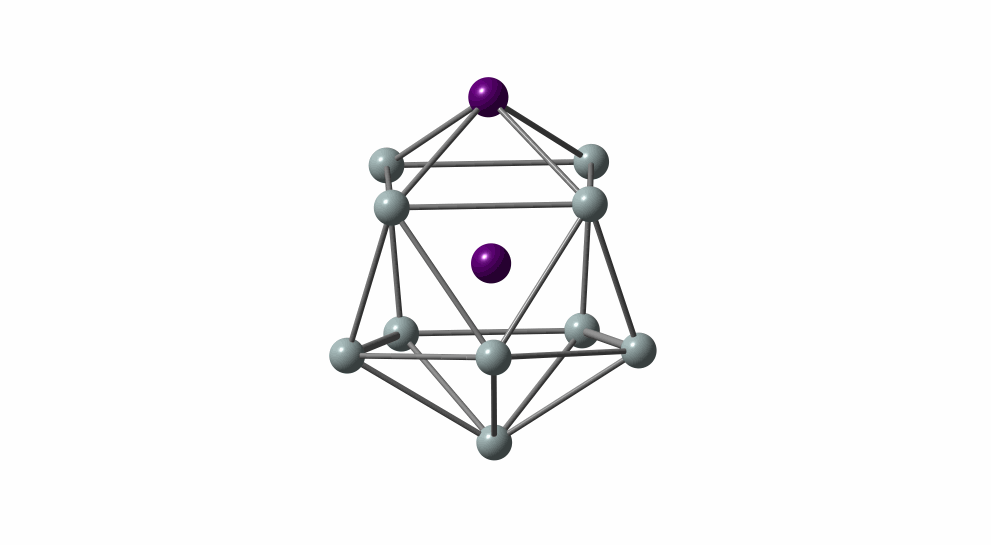

Supplement: Supplementary file 3 — jp1c10027_si_003.zip [file jp1c10027_si_003.zip › Mn2Si10_gifs/186_a`.gif]

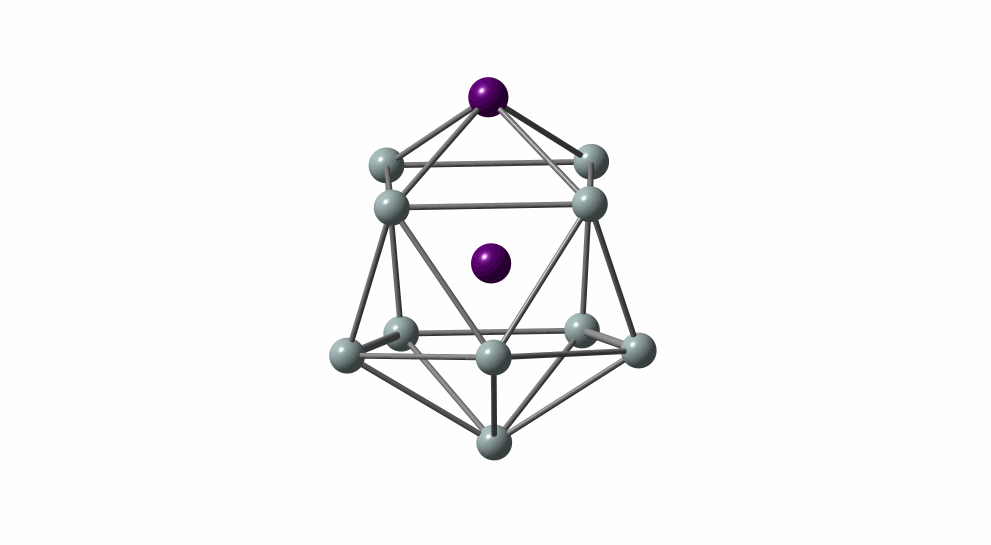

Supplement: Supplementary file 3 — jp1c10027_si_003.zip [file jp1c10027_si_003.zip › Mn2Si10_gifs/186_a``.gif]

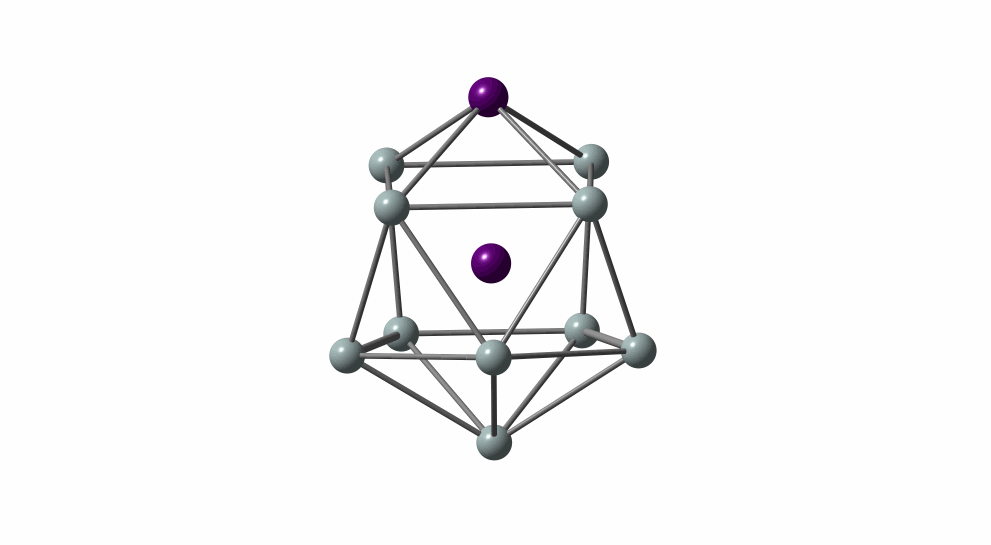

Supplement: Supplementary file 3 — jp1c10027_si_003.zip [file jp1c10027_si_003.zip › Mn2Si10_gifs/187_a``.gif]

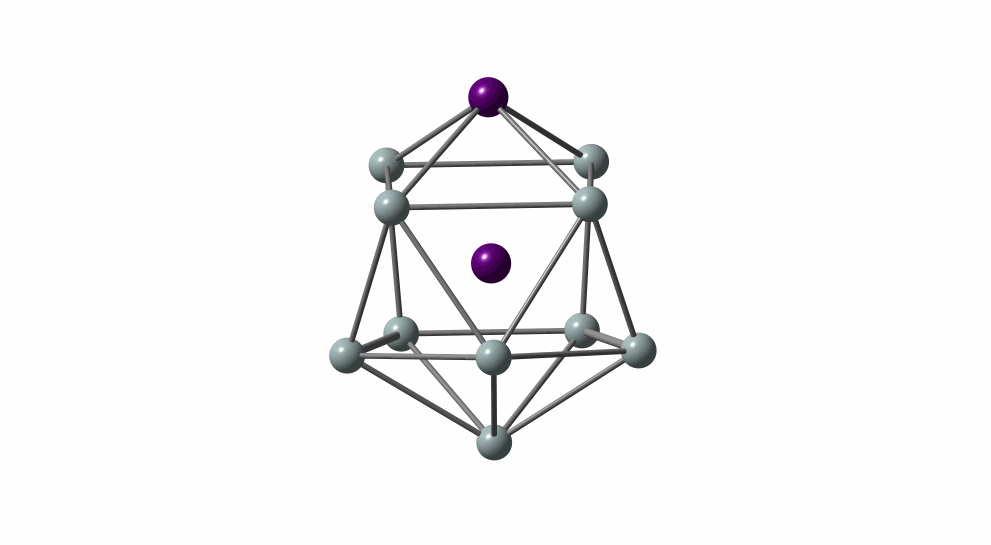

Supplement: Supplementary file 3 — jp1c10027_si_003.zip [file jp1c10027_si_003.zip › Mn2Si10_gifs/203_a`.gif]

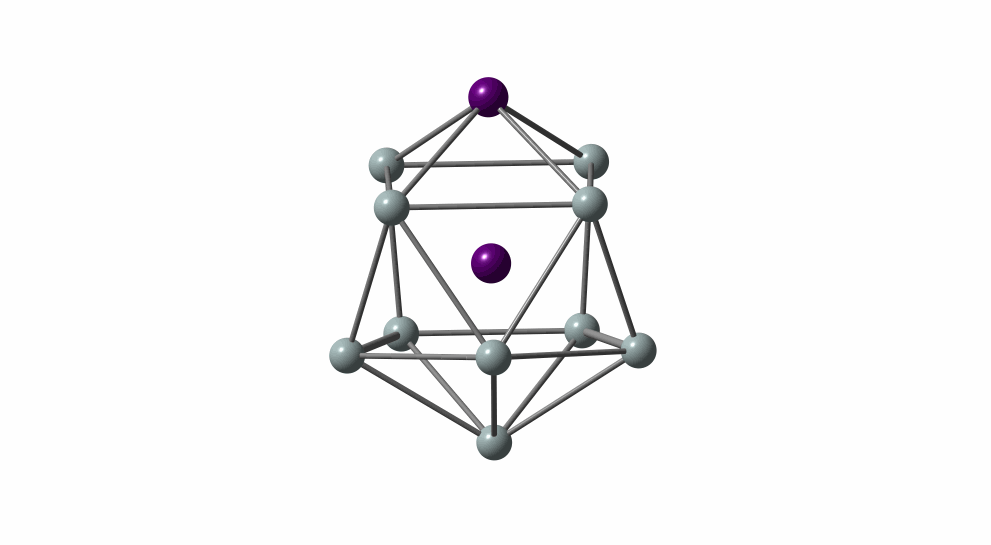

Supplement: Supplementary file 3 — jp1c10027_si_003.zip [file jp1c10027_si_003.zip › Mn2Si10_gifs/216_a`.gif]

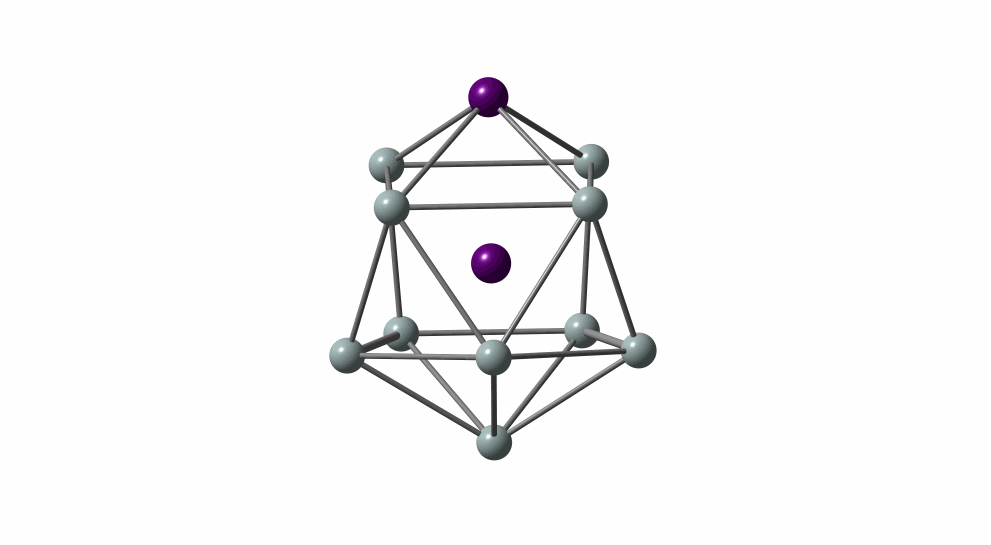

Supplement: Supplementary file 3 — jp1c10027_si_003.zip [file jp1c10027_si_003.zip › Mn2Si10_gifs/219_a``.gif]

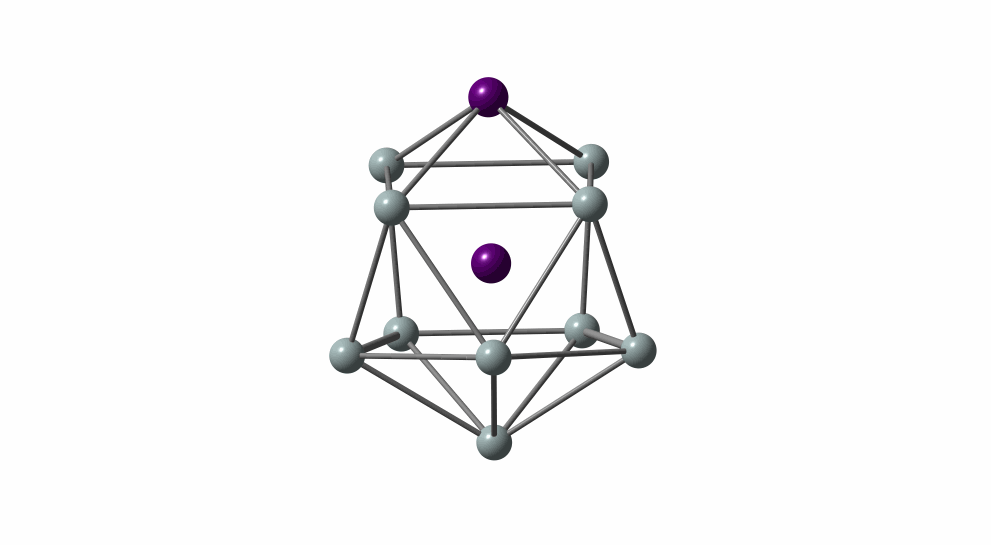

Supplement: Supplementary file 3 — jp1c10027_si_003.zip [file jp1c10027_si_003.zip › Mn2Si10_gifs/229_a``.gif]

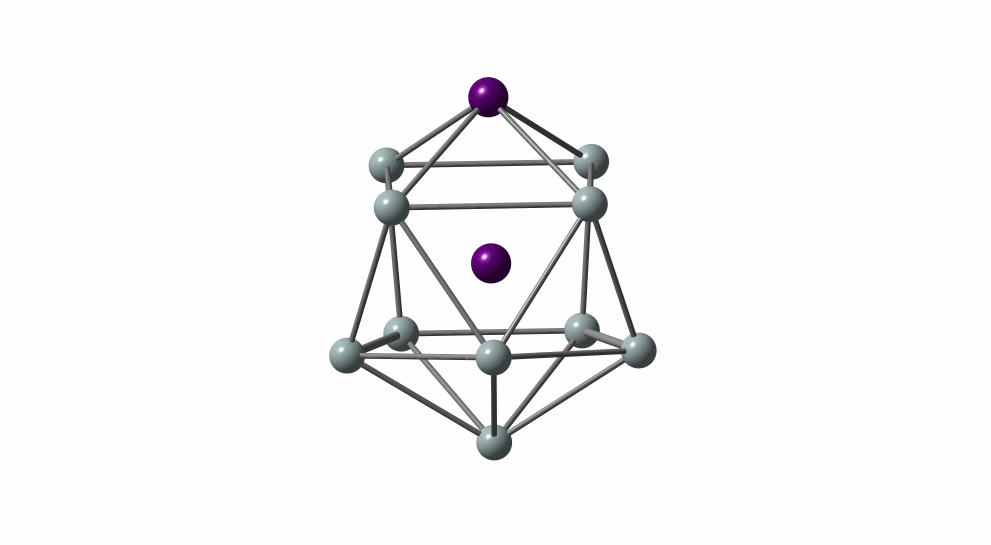

Supplement: Supplementary file 3 — jp1c10027_si_003.zip [file jp1c10027_si_003.zip › Mn2Si10_gifs/245_a`.gif]

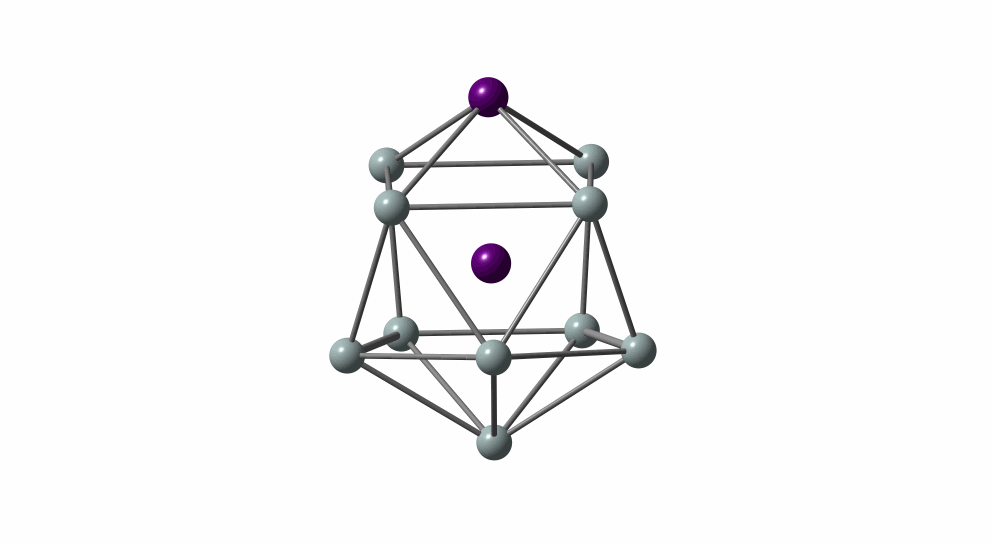

Supplement: Supplementary file 3 — jp1c10027_si_003.zip [file jp1c10027_si_003.zip › Mn2Si10_gifs/263_a`.gif]

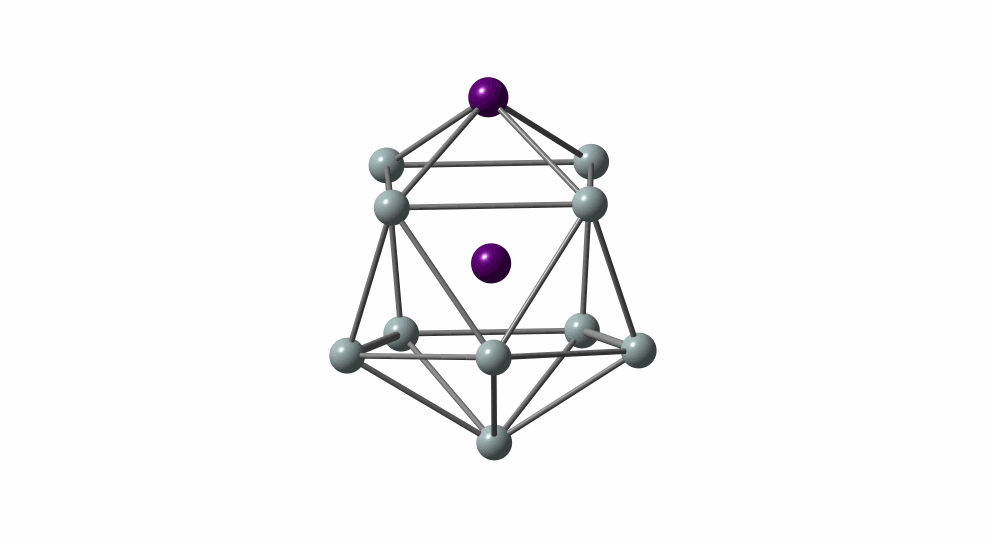

Supplement: Supplementary file 3 — jp1c10027_si_003.zip [file jp1c10027_si_003.zip › Mn2Si10_gifs/275_a`.gif]

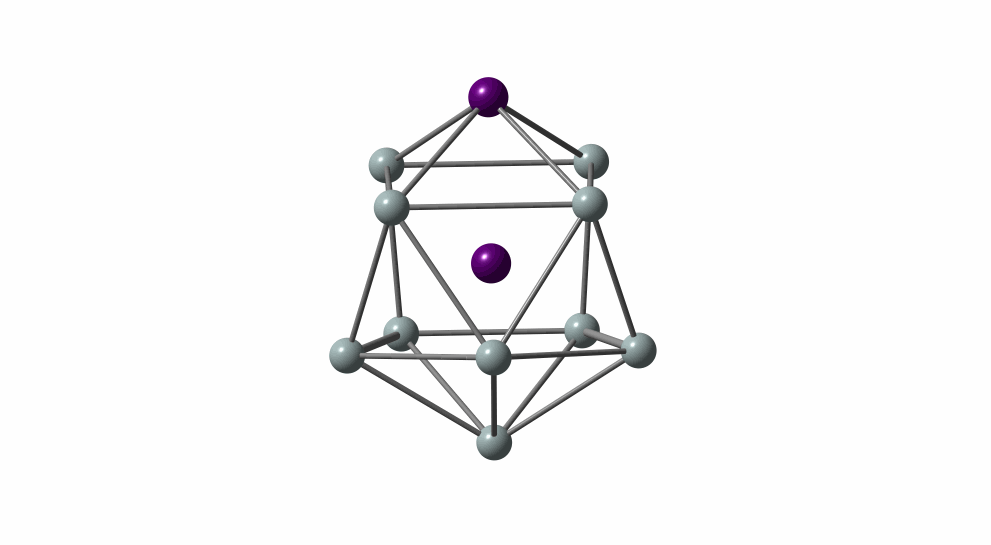

Supplement: Supplementary file 3 — jp1c10027_si_003.zip [file jp1c10027_si_003.zip › Mn2Si10_gifs/316_a`.gif]

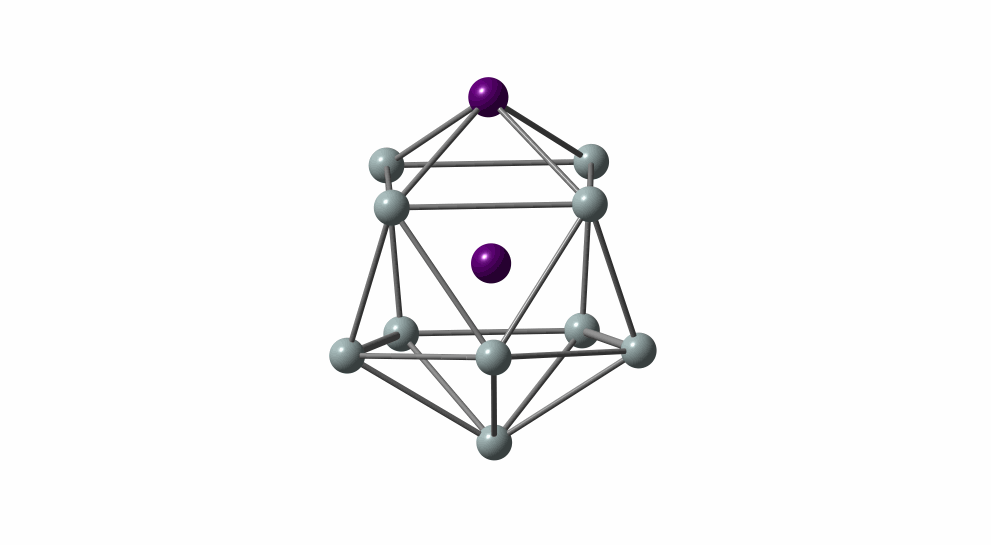

Supplement: Supplementary file 3 — jp1c10027_si_003.zip [file jp1c10027_si_003.zip › Mn2Si10_gifs/347_a`.gif]

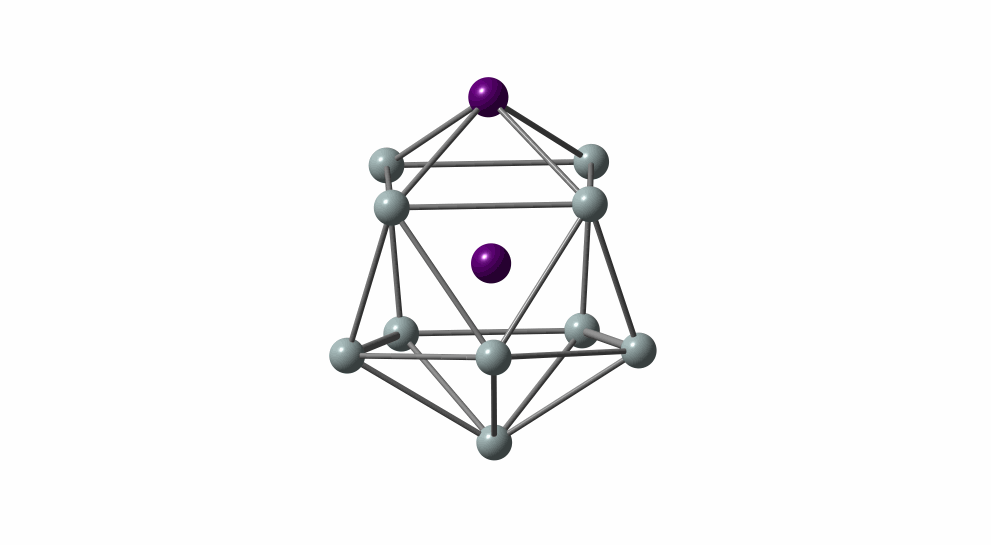

Supplement: Supplementary file 3 — jp1c10027_si_003.zip [file jp1c10027_si_003.zip › Mn2Si10_gifs/430_a``.gif]

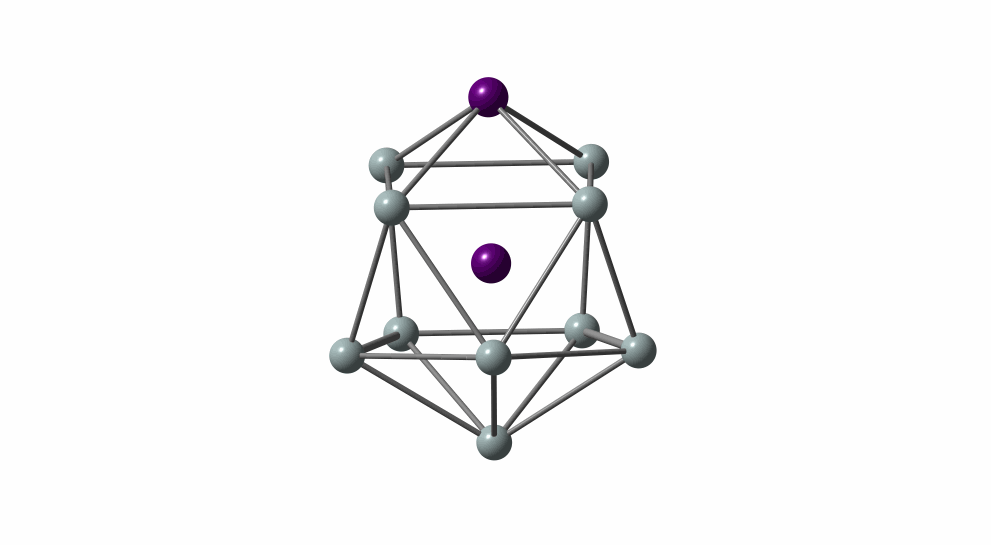

Supplement: Supplementary file 3 — jp1c10027_si_003.zip [file jp1c10027_si_003.zip › Mn2Si10_gifs/448_a`.gif]

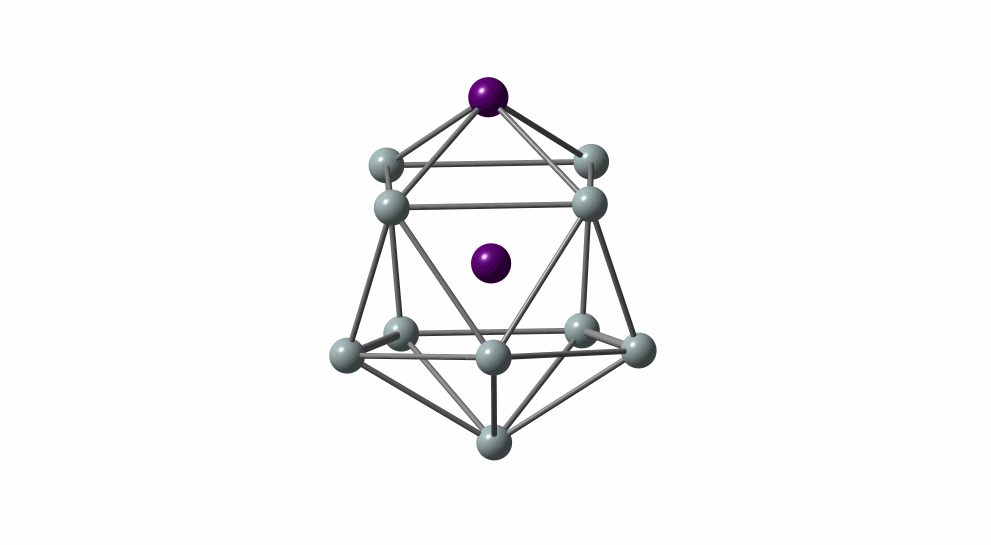

Supplement: Supplementary file 3 — jp1c10027_si_003.zip [file jp1c10027_si_003.zip › Mn2Si10_gifs/462_a`.gif]

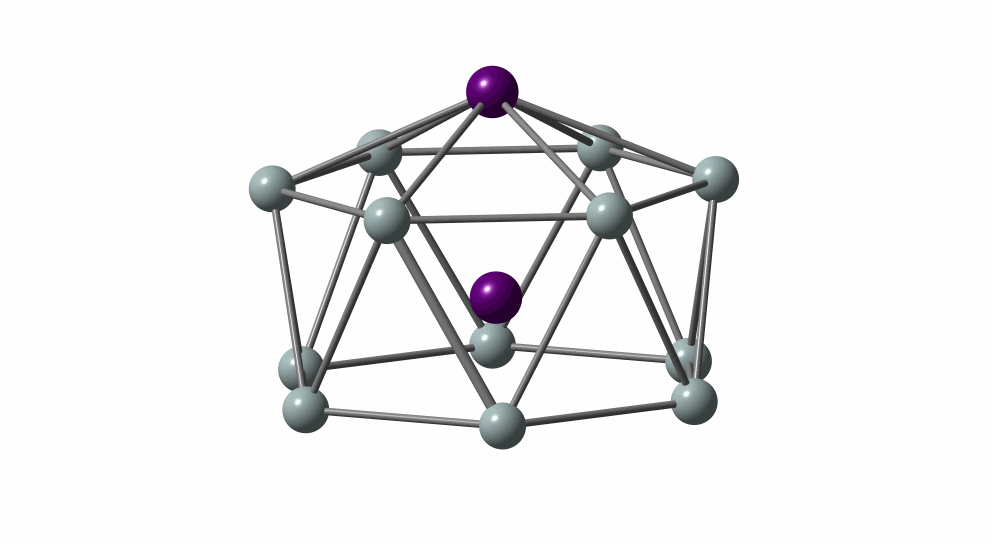

Supplement: Supplementary file 3 — jp1c10027_si_003.zip [file jp1c10027_si_003.zip › Mn2Si12_gifs/198_e1.gif]

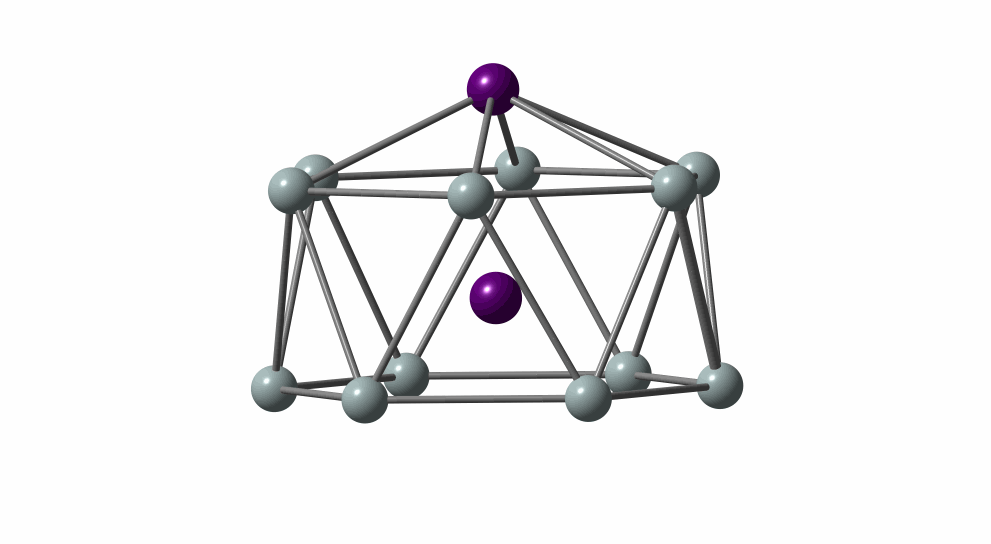

Supplement: Supplementary file 3 — jp1c10027_si_003.zip [file jp1c10027_si_003.zip › Mn2Si12_gifs/203_a1.gif]

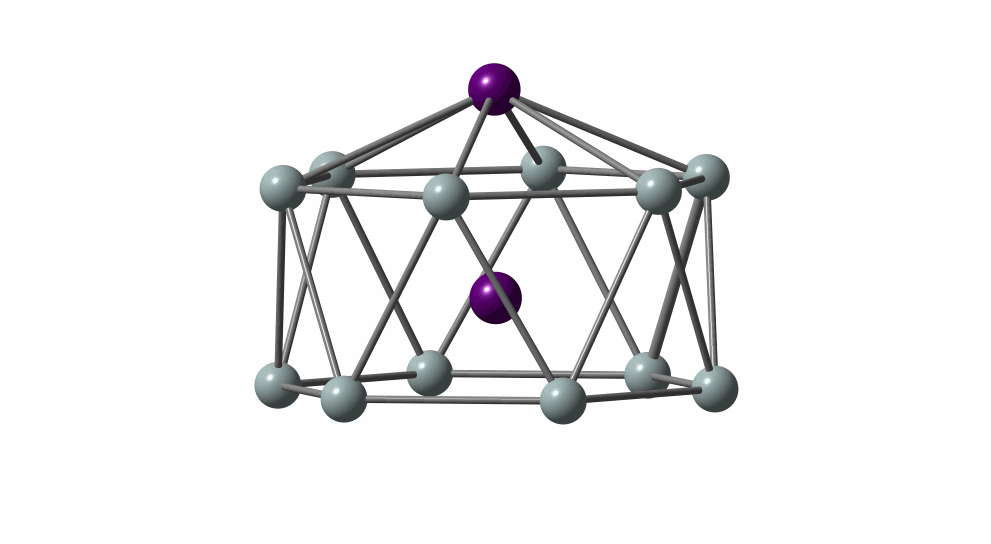

Supplement: Supplementary file 3 — jp1c10027_si_003.zip [file jp1c10027_si_003.zip › Mn2Si12_gifs/239_e1.gif]

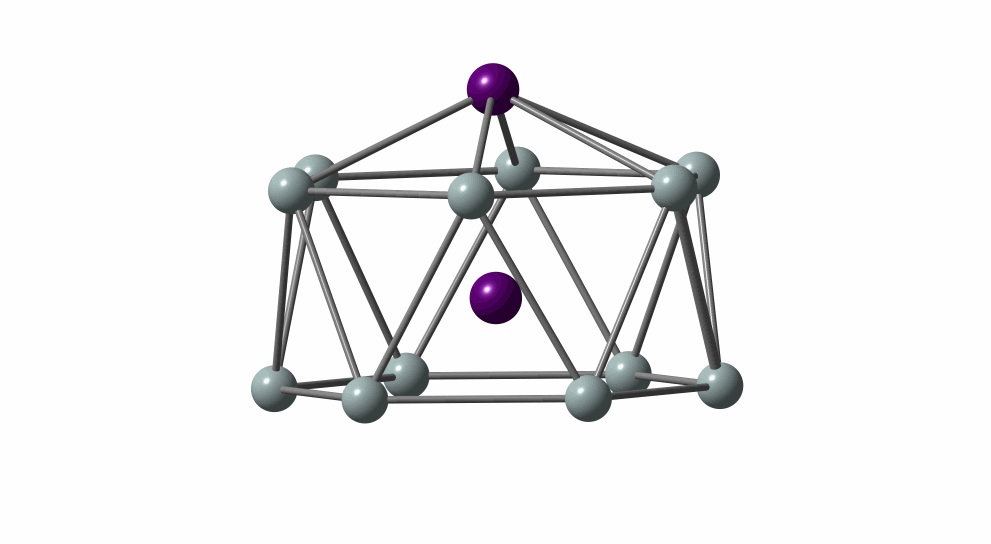

Supplement: Supplementary file 3 — jp1c10027_si_003.zip [file jp1c10027_si_003.zip › Mn2Si12_gifs/270_a1.gif]

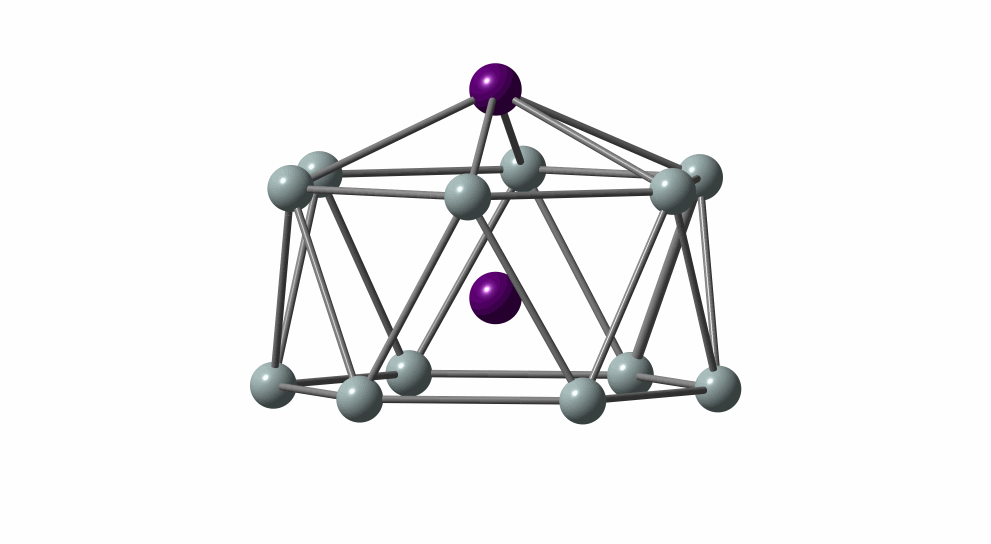

Supplement: Supplementary file 3 — jp1c10027_si_003.zip [file jp1c10027_si_003.zip › Mn2Si12_gifs/272_e1.gif]

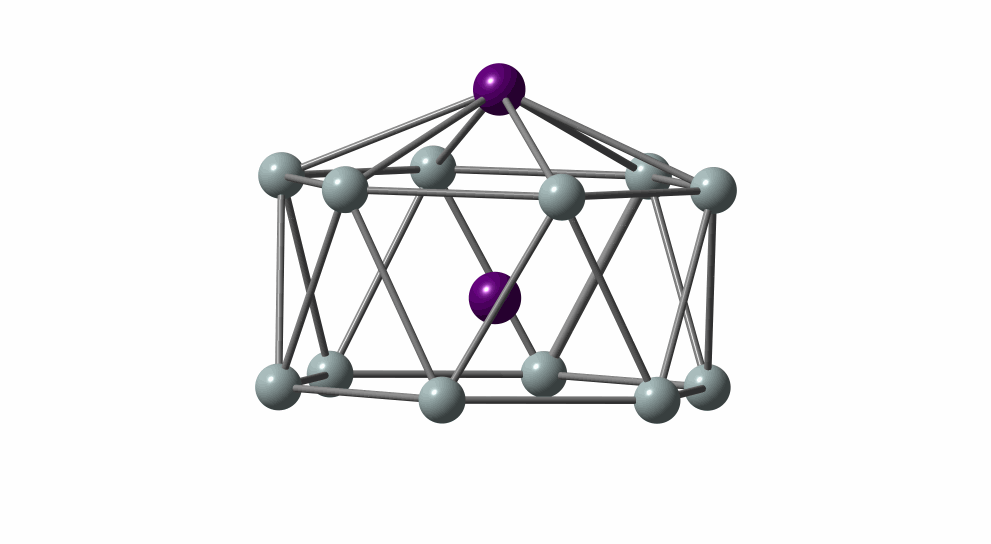

Supplement: Supplementary file 3 — jp1c10027_si_003.zip [file jp1c10027_si_003.zip › Mn2Si12_gifs/288_e1.gif]

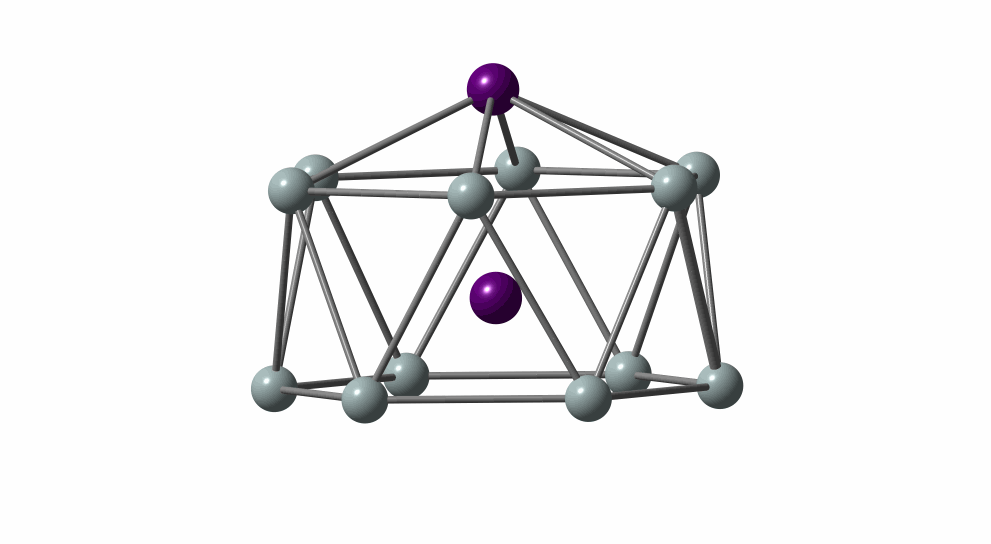

Supplement: Supplementary file 3 — jp1c10027_si_003.zip [file jp1c10027_si_003.zip › Mn2Si12_gifs/317_a1.gif]

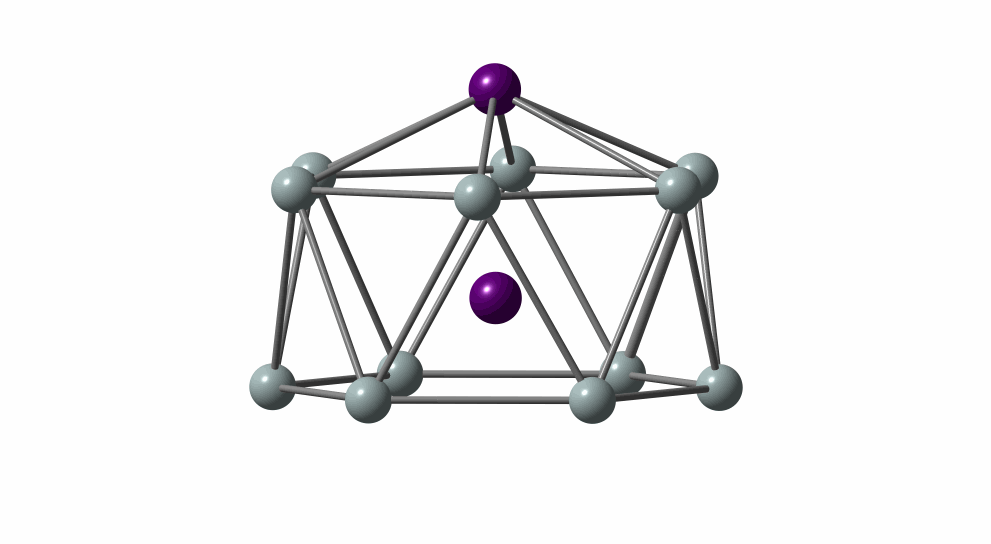

Supplement: Supplementary file 3 — jp1c10027_si_003.zip [file jp1c10027_si_003.zip › Mn2Si12_gifs/330_e1.gif]

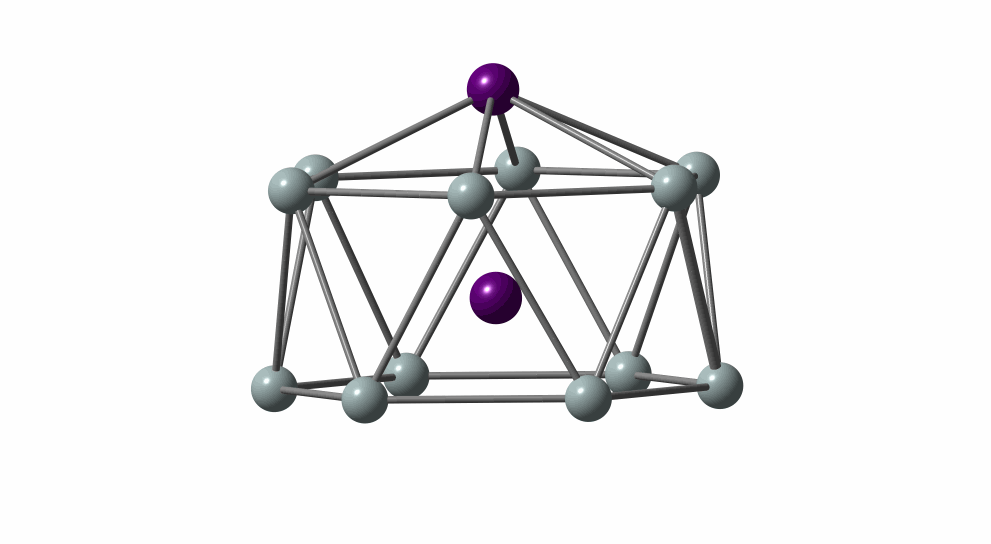

Supplement: Supplementary file 3 — jp1c10027_si_003.zip [file jp1c10027_si_003.zip › Mn2Si12_gifs/345_a1.gif]

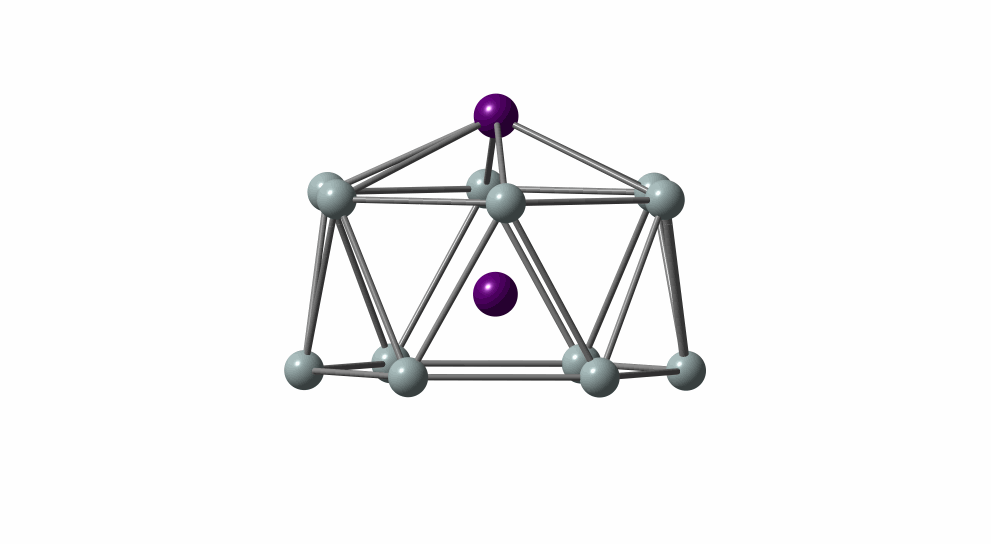

Supplement: Supplementary file 3 — jp1c10027_si_003.zip [file jp1c10027_si_003.zip › Mn2Si12_gifs/364_e1.gif]

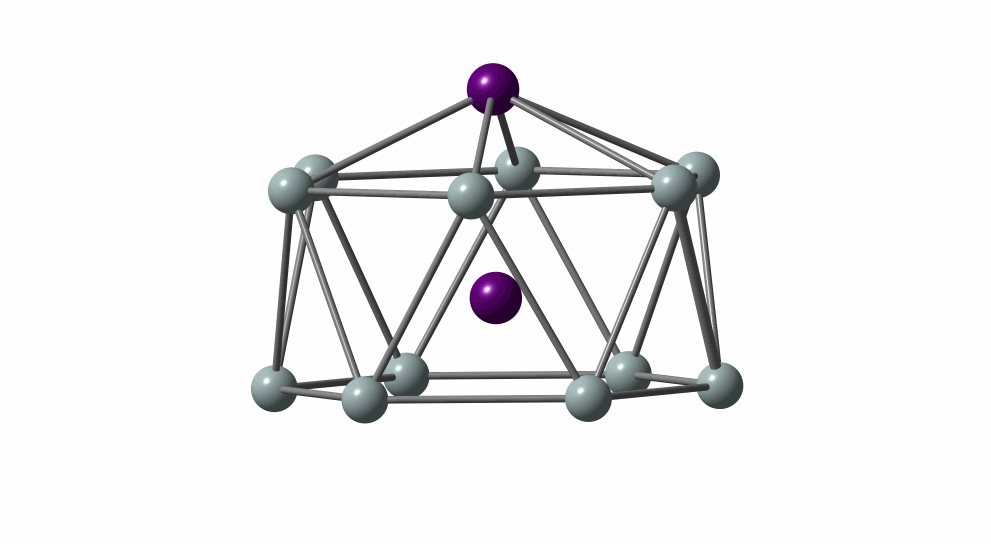

Supplement: Supplementary file 3 — jp1c10027_si_003.zip [file jp1c10027_si_003.zip › Mn2Si12_gifs/385_a1.gif]

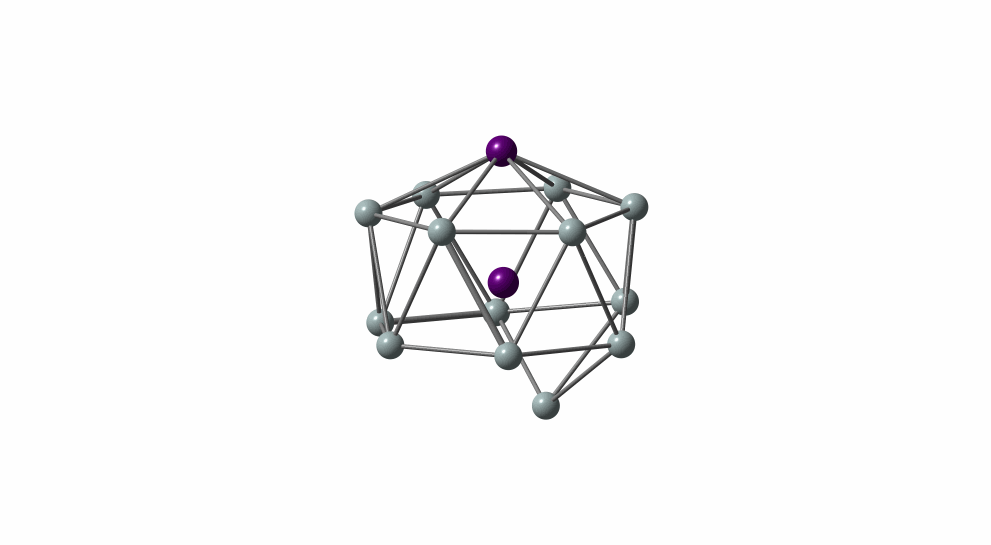

Supplement: Supplementary file 3 — jp1c10027_si_003.zip [file jp1c10027_si_003.zip › Mn2Si13_gifs/238.gif]

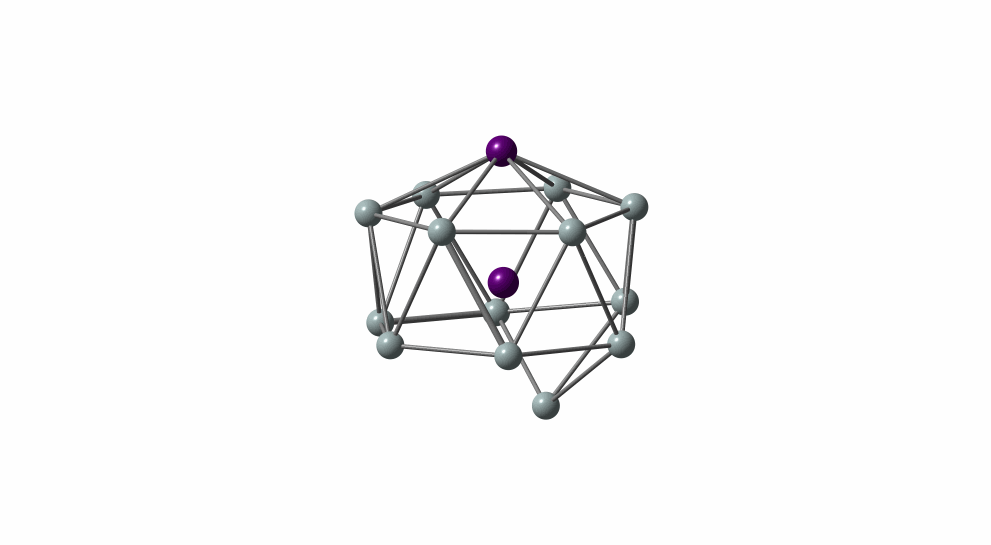

Supplement: Supplementary file 3 — jp1c10027_si_003.zip [file jp1c10027_si_003.zip › Mn2Si13_gifs/240.gif]

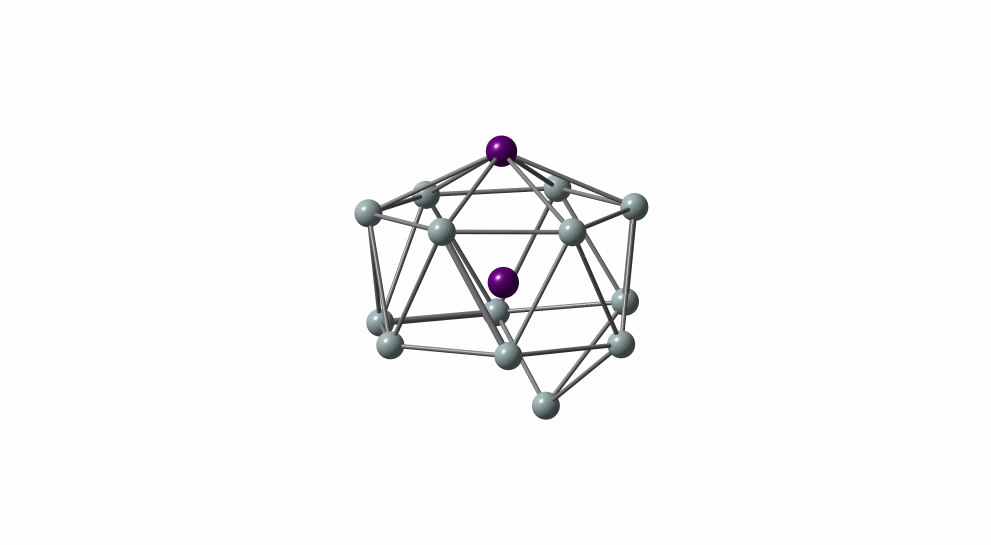

Supplement: Supplementary file 3 — jp1c10027_si_003.zip [file jp1c10027_si_003.zip › Mn2Si13_gifs/257.gif]

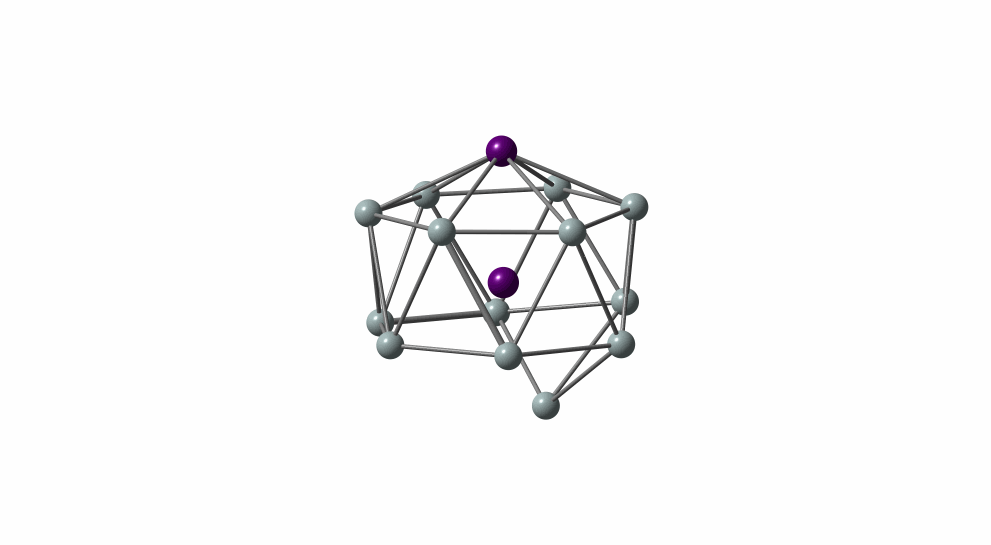

Supplement: Supplementary file 3 — jp1c10027_si_003.zip [file jp1c10027_si_003.zip › Mn2Si13_gifs/278.gif]

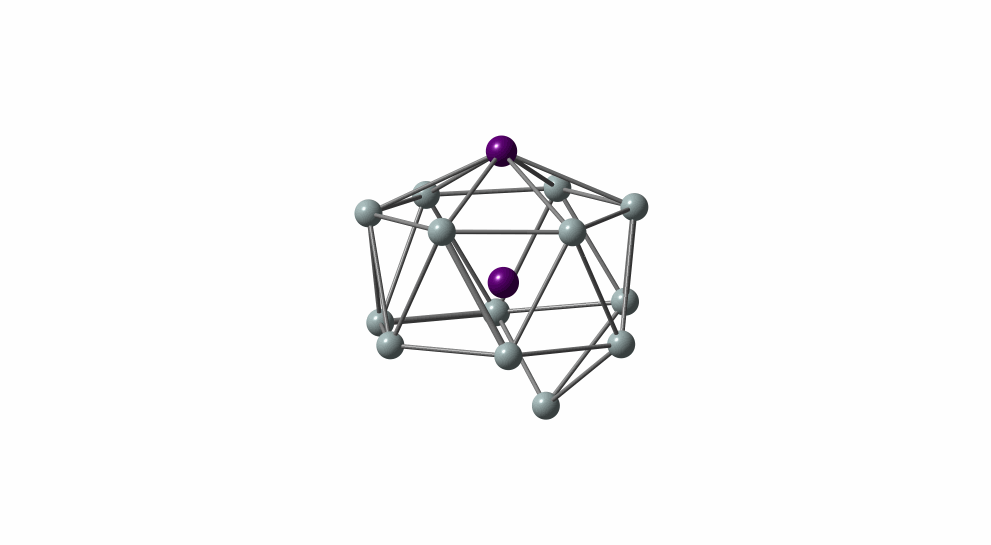

Supplement: Supplementary file 3 — jp1c10027_si_003.zip [file jp1c10027_si_003.zip › Mn2Si13_gifs/281.gif]

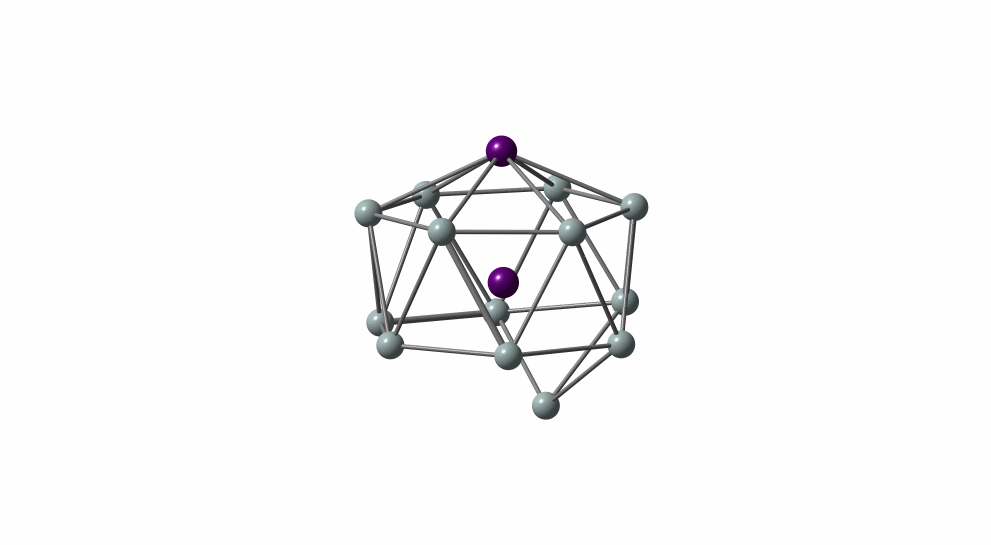

Supplement: Supplementary file 3 — jp1c10027_si_003.zip [file jp1c10027_si_003.zip › Mn2Si13_gifs/289.gif]

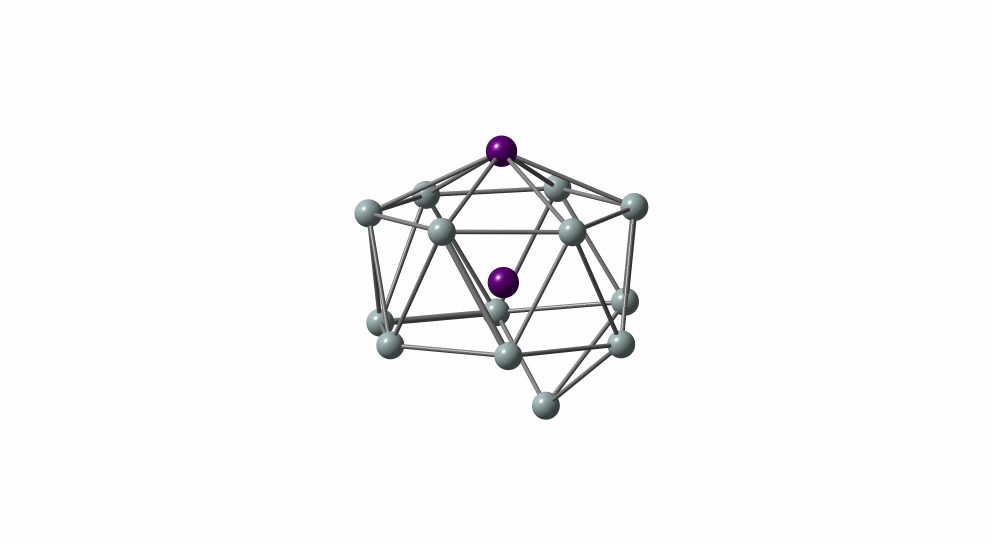

Supplement: Supplementary file 3 — jp1c10027_si_003.zip [file jp1c10027_si_003.zip › Mn2Si13_gifs/298.gif]

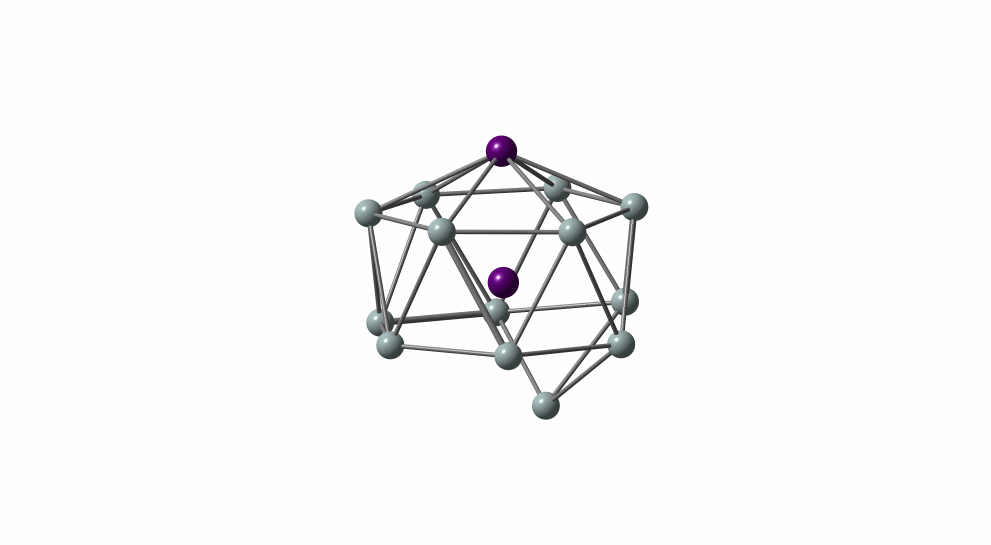

Supplement: Supplementary file 3 — jp1c10027_si_003.zip [file jp1c10027_si_003.zip › Mn2Si13_gifs/311.gif]

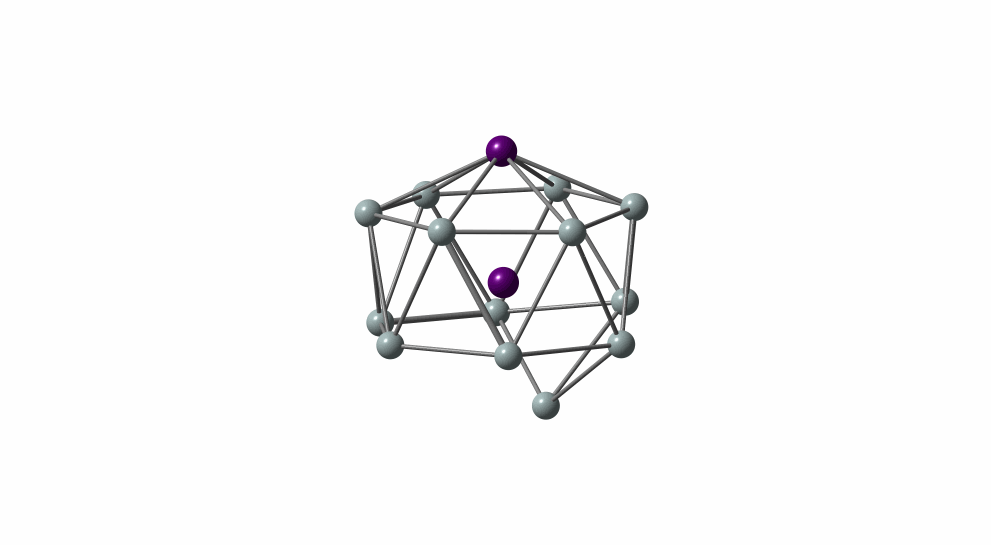

Supplement: Supplementary file 3 — jp1c10027_si_003.zip [file jp1c10027_si_003.zip › Mn2Si13_gifs/315.gif]

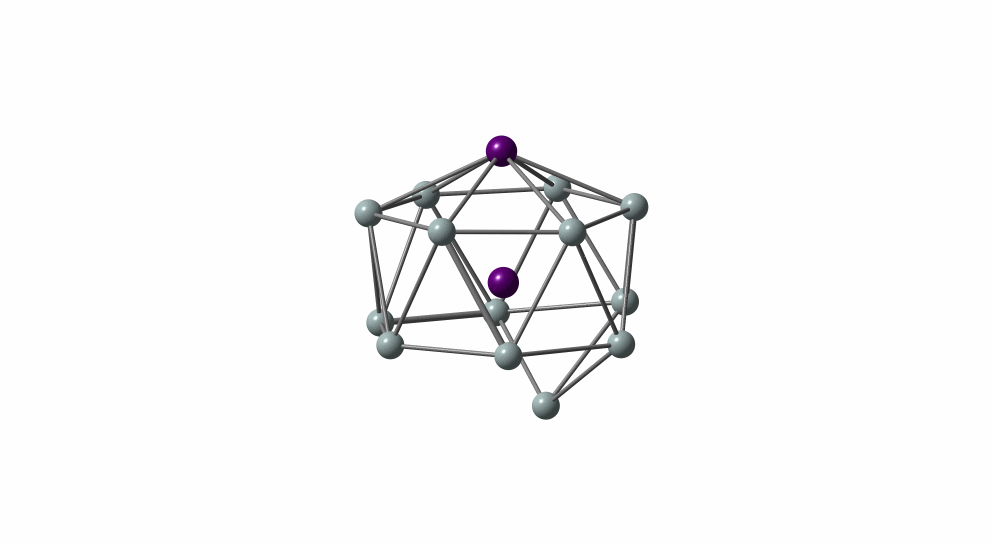

Supplement: Supplementary file 3 — jp1c10027_si_003.zip [file jp1c10027_si_003.zip › Mn2Si13_gifs/326.gif]

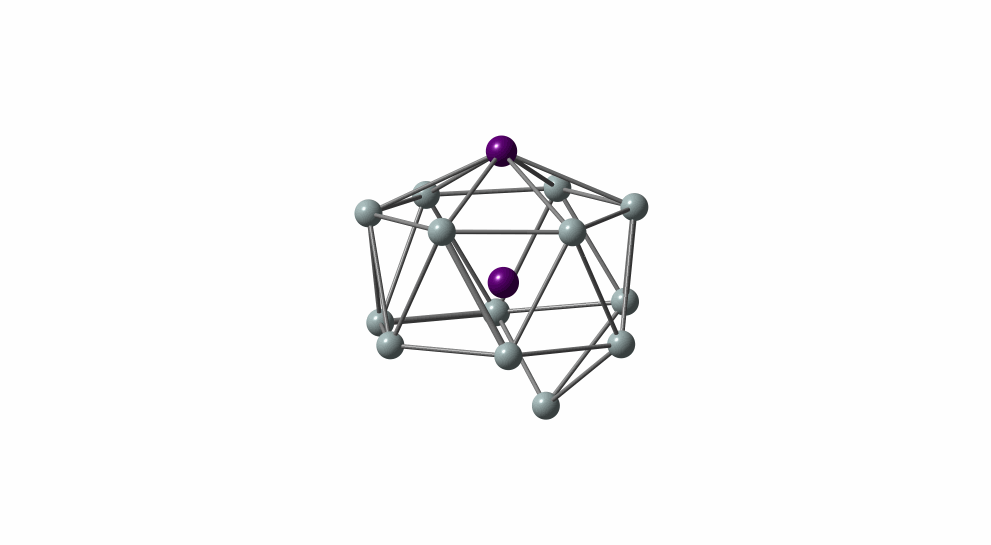

Supplement: Supplementary file 3 — jp1c10027_si_003.zip [file jp1c10027_si_003.zip › Mn2Si13_gifs/349.gif]

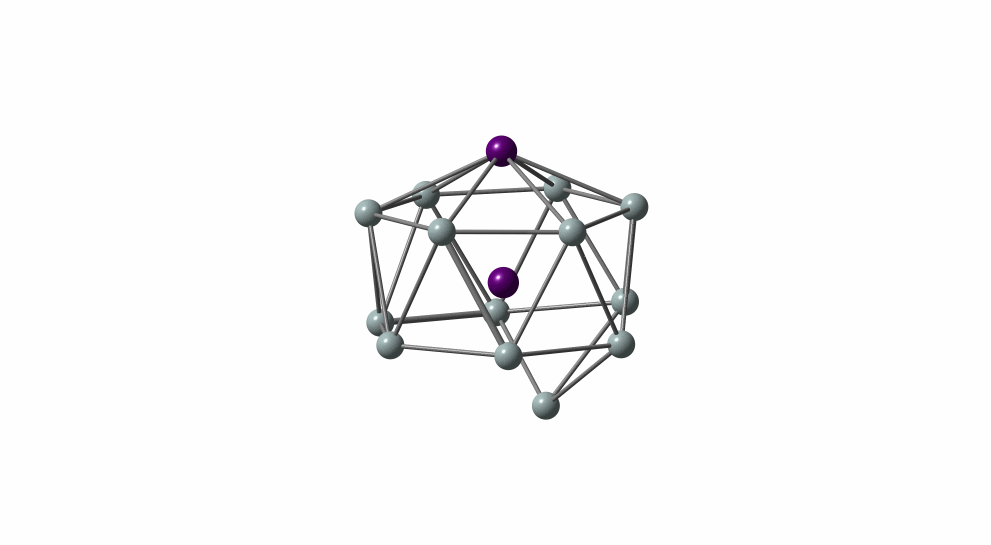

Supplement: Supplementary file 3 — jp1c10027_si_003.zip [file jp1c10027_si_003.zip › Mn2Si13_gifs/350.gif]

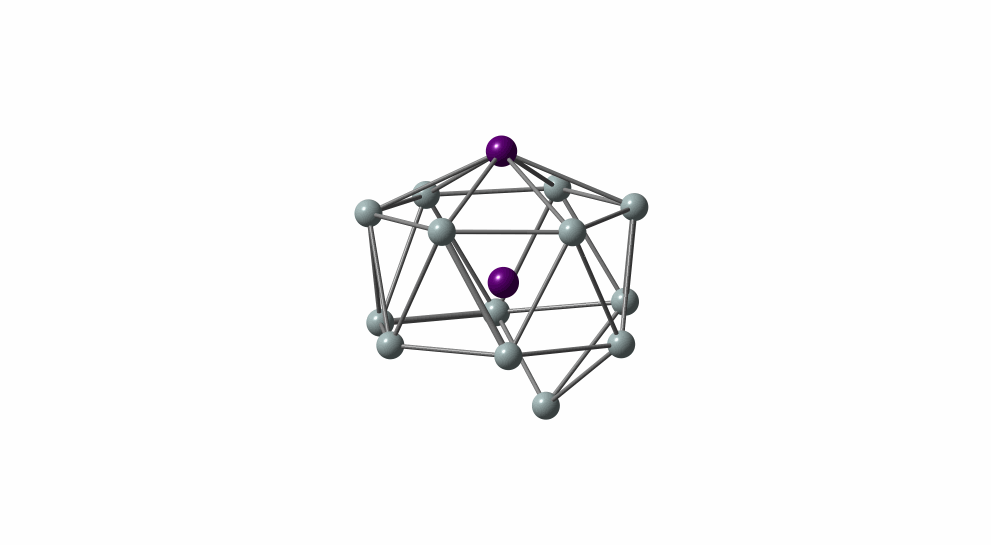

Supplement: Supplementary file 3 — jp1c10027_si_003.zip [file jp1c10027_si_003.zip › Mn2Si13_gifs/373.gif]

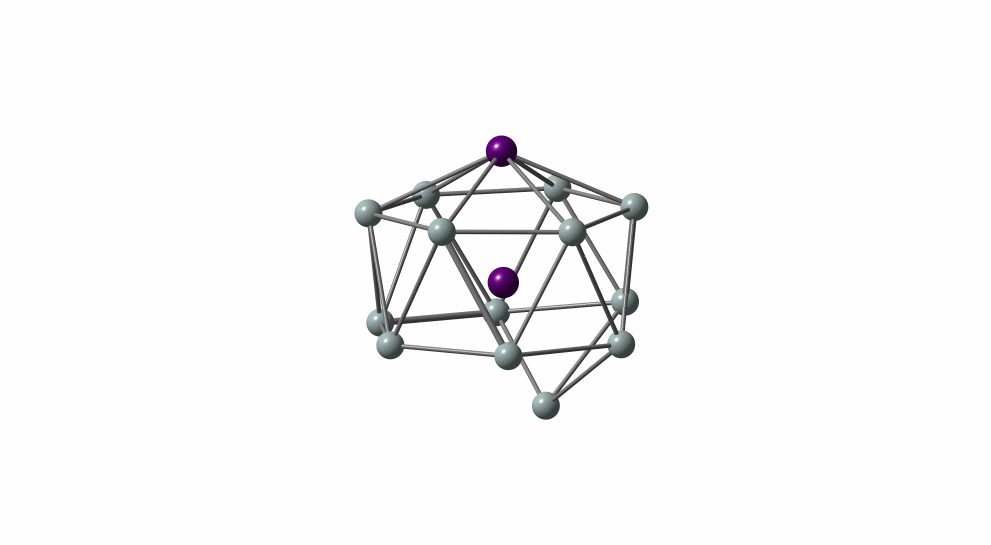

Supplement: Supplementary file 3 — jp1c10027_si_003.zip [file jp1c10027_si_003.zip › Mn2Si13_gifs/388.gif]

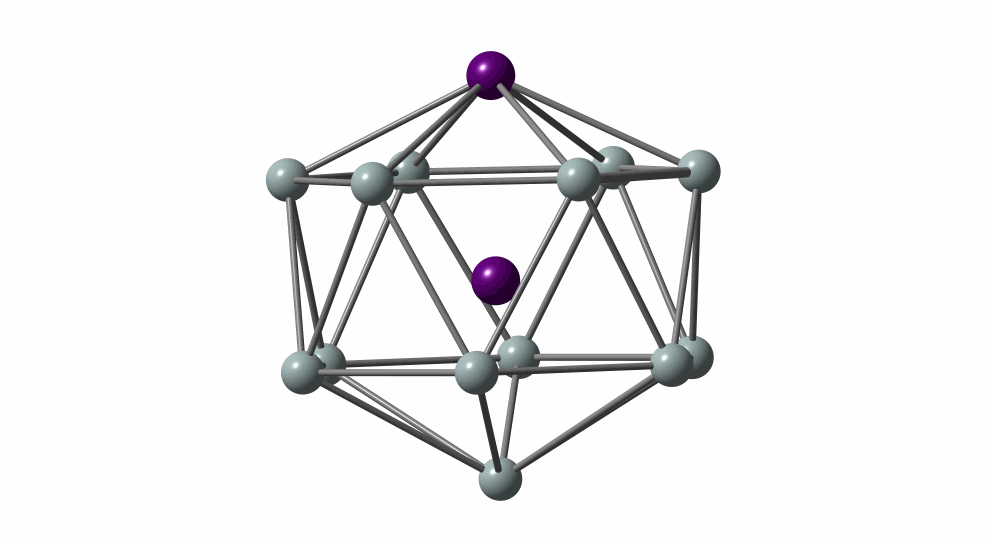

Supplement: Supplementary file 3 — jp1c10027_si_003.zip [file jp1c10027_si_003.zip › Mn2Si13+_gifs/158_e1.gif]

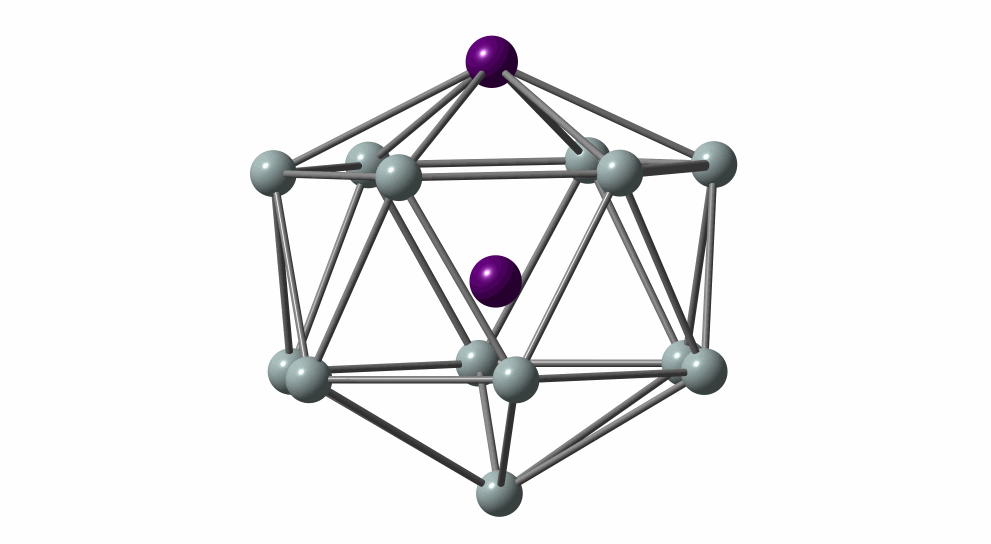

Supplement: Supplementary file 3 — jp1c10027_si_003.zip [file jp1c10027_si_003.zip › Mn2Si13+_gifs/196_a1.gif]

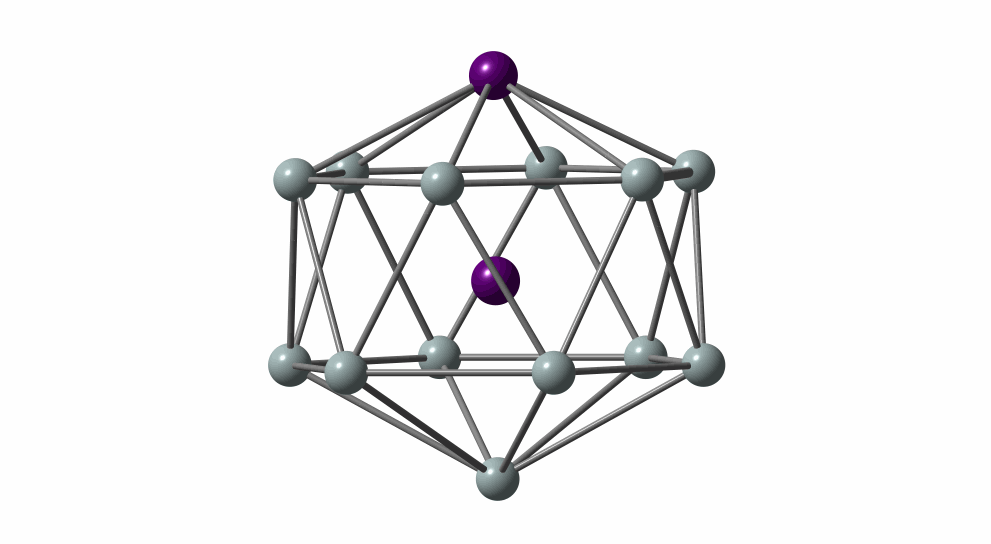

Supplement: Supplementary file 3 — jp1c10027_si_003.zip [file jp1c10027_si_003.zip › Mn2Si13+_gifs/224_e1.gif]

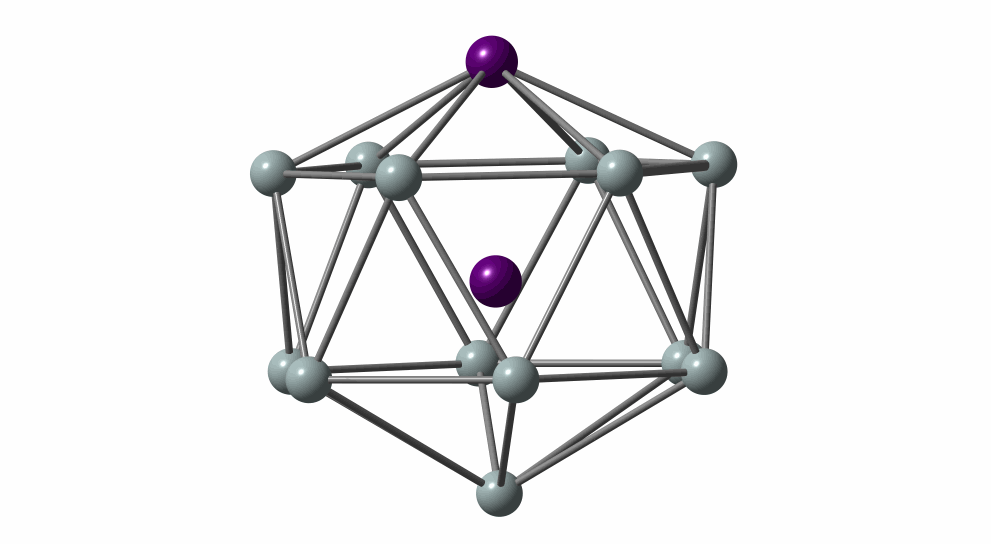

Supplement: Supplementary file 3 — jp1c10027_si_003.zip [file jp1c10027_si_003.zip › Mn2Si13+_gifs/256_a1.gif]

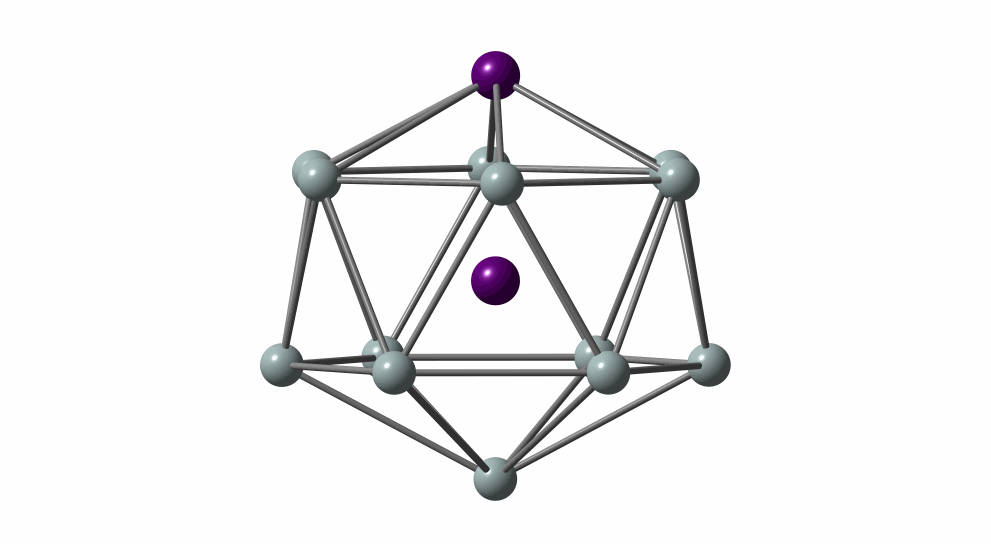

Supplement: Supplementary file 3 — jp1c10027_si_003.zip [file jp1c10027_si_003.zip › Mn2Si13+_gifs/268_e1.gif]

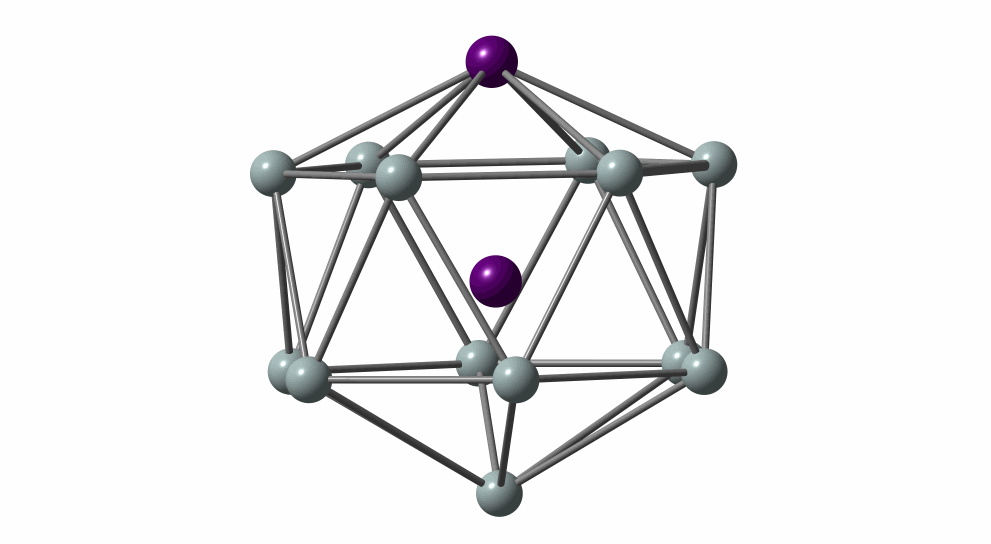

Supplement: Supplementary file 3 — jp1c10027_si_003.zip [file jp1c10027_si_003.zip › Mn2Si13+_gifs/286_a1.gif]

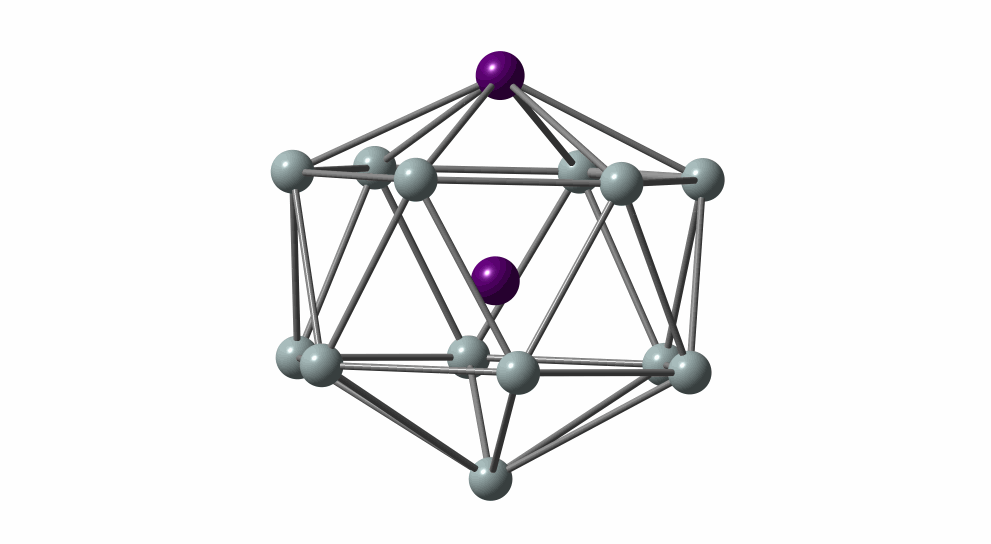

Supplement: Supplementary file 3 — jp1c10027_si_003.zip [file jp1c10027_si_003.zip › Mn2Si13+_gifs/310_e1.gif]

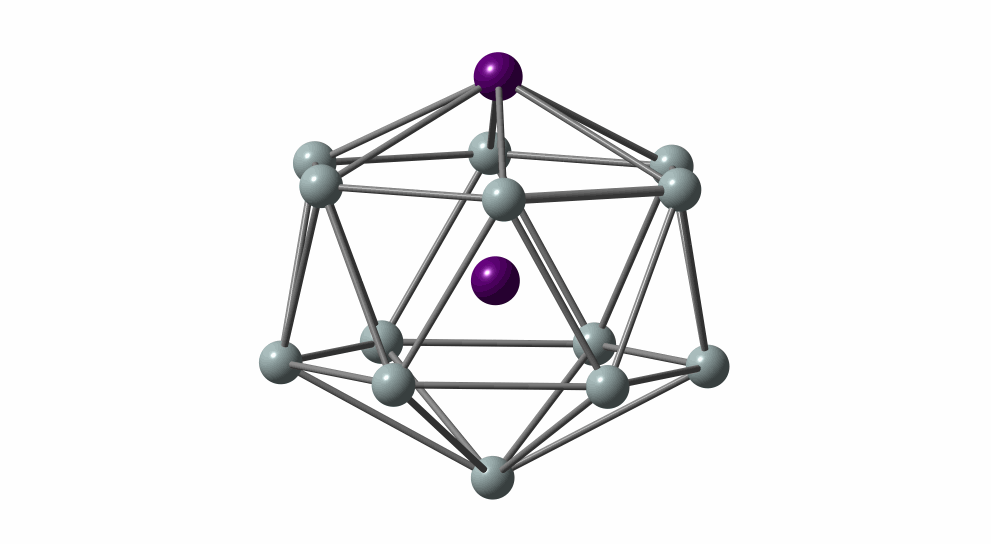

Supplement: Supplementary file 3 — jp1c10027_si_003.zip [file jp1c10027_si_003.zip › Mn2Si13+_gifs/315_e1.gif]

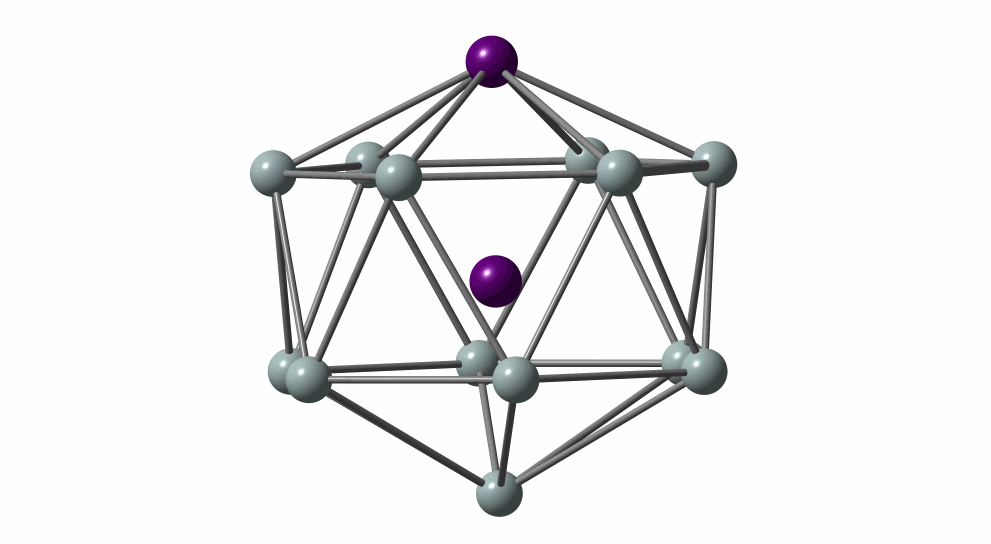

Supplement: Supplementary file 3 — jp1c10027_si_003.zip [file jp1c10027_si_003.zip › Mn2Si13+_gifs/319_a1.gif]

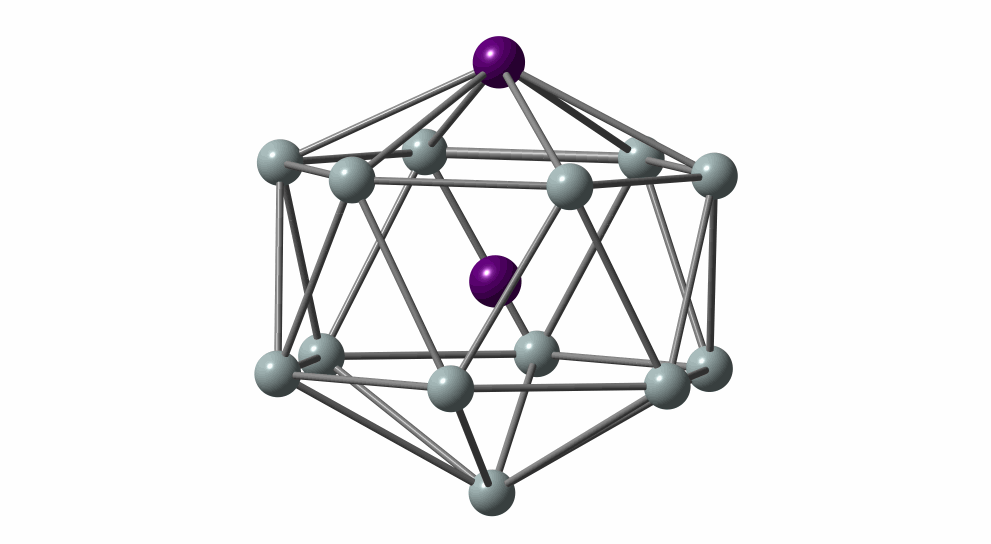

Supplement: Supplementary file 3 — jp1c10027_si_003.zip [file jp1c10027_si_003.zip › Mn2Si13+_gifs/359_e1.gif]

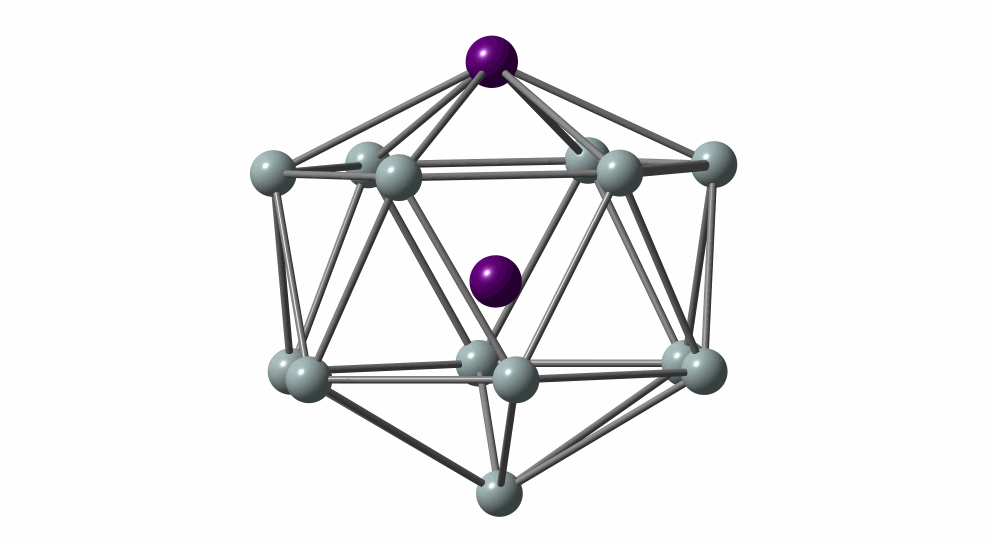

Supplement: Supplementary file 3 — jp1c10027_si_003.zip [file jp1c10027_si_003.zip › Mn2Si13+_gifs/373_a1.gif]

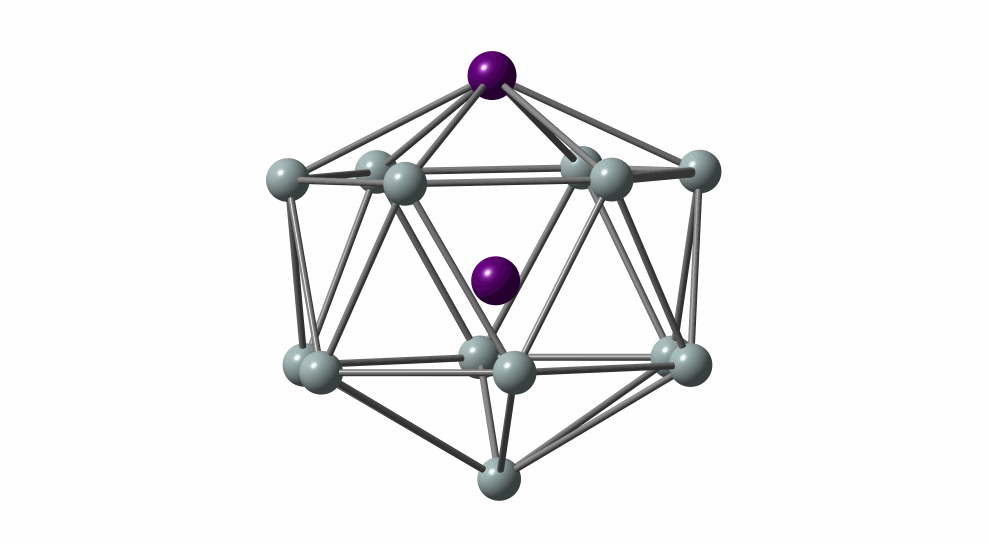

Supplement: Supplementary file 3 — jp1c10027_si_003.zip [file jp1c10027_si_003.zip › Mn2Si13+_gifs/419_a1.gif]

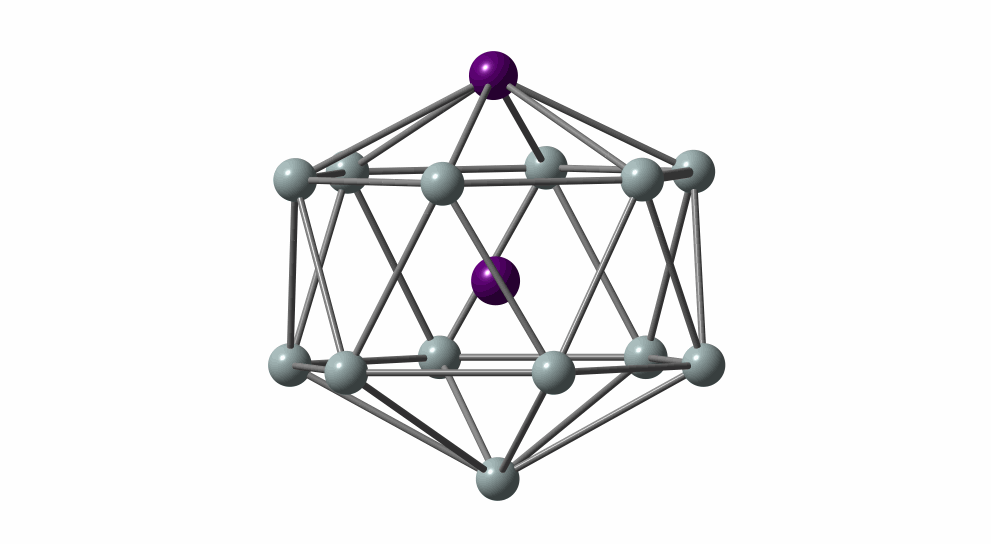

Supplement: Supplementary file 3 — jp1c10027_si_003.zip [file jp1c10027_si_003.zip › Mn2Si13+_gifs/43_e1.gif]

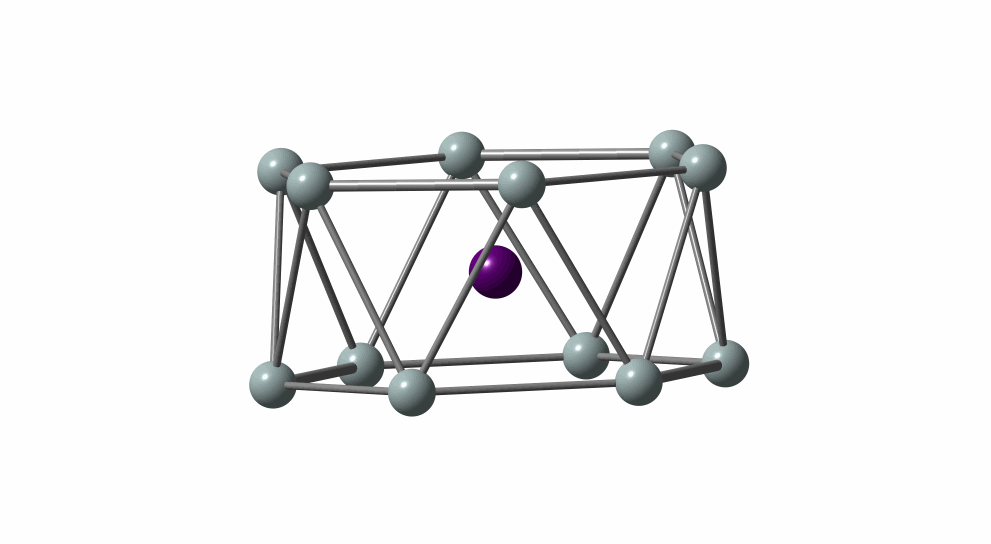

Supplement: Supplementary file 3 — jp1c10027_si_003.zip [file jp1c10027_si_003.zip › MnSi12+_gifs/190_e1.gif]

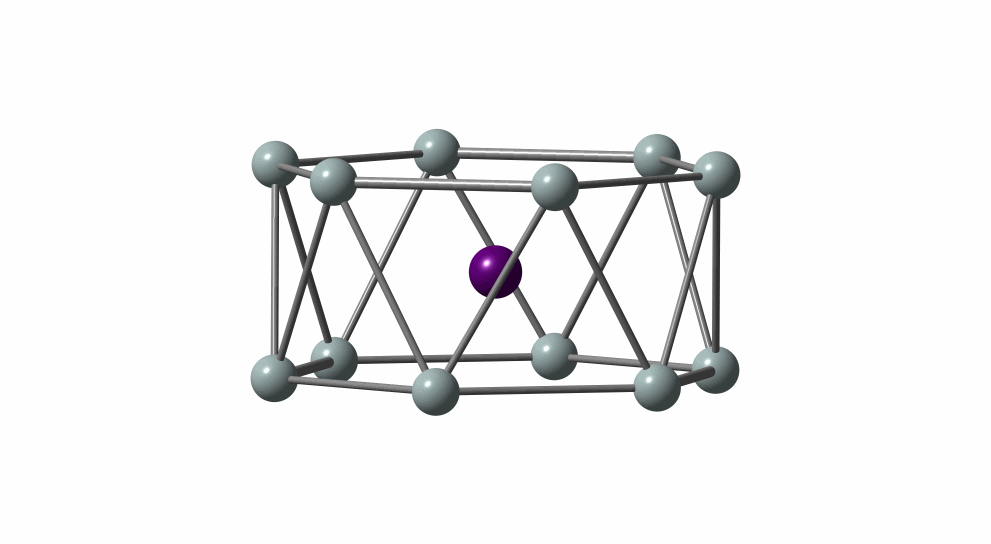

Supplement: Supplementary file 3 — jp1c10027_si_003.zip [file jp1c10027_si_003.zip › MnSi12+_gifs/200_e5.gif]

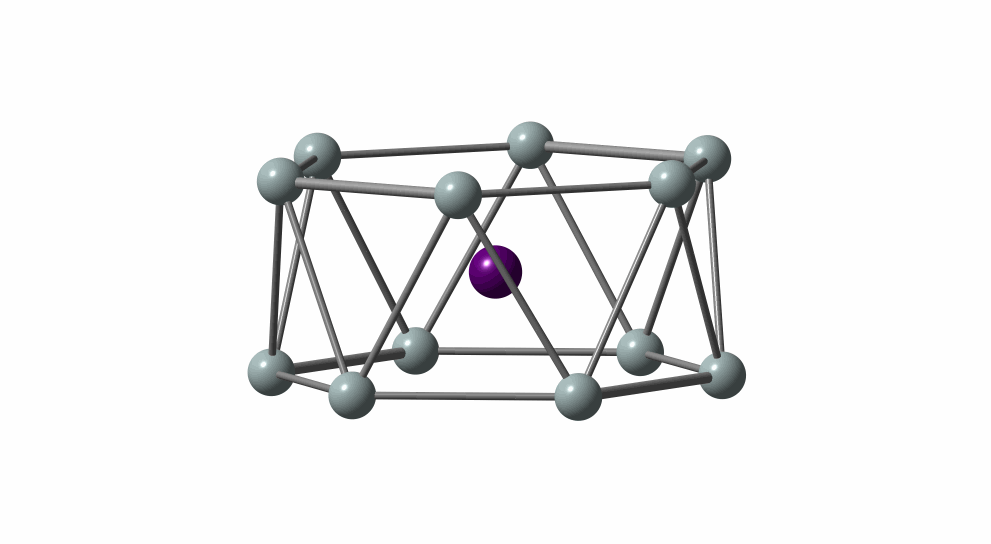

Supplement: Supplementary file 3 — jp1c10027_si_003.zip [file jp1c10027_si_003.zip › MnSi12+_gifs/219_b2.gif]

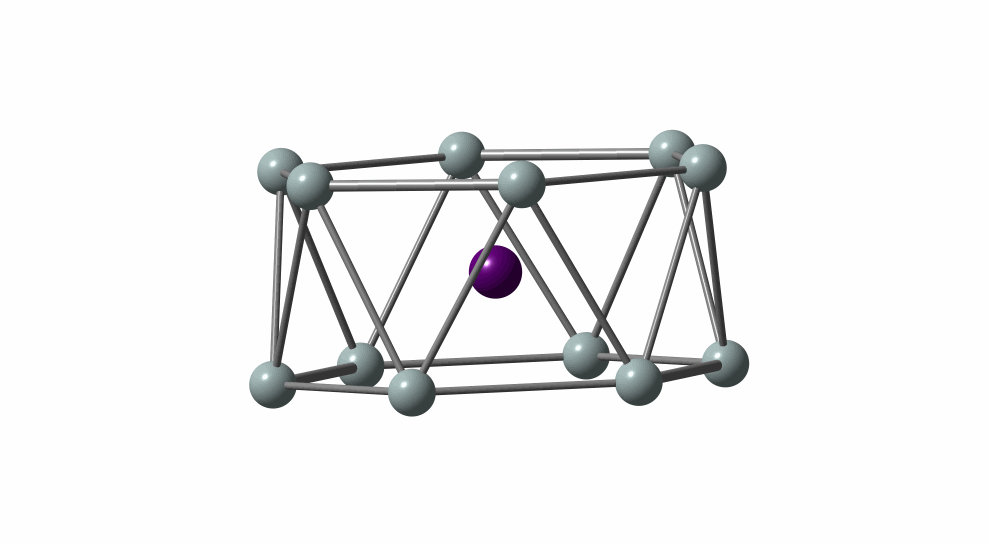

Supplement: Supplementary file 3 — jp1c10027_si_003.zip [file jp1c10027_si_003.zip › MnSi12+_gifs/277_e1.gif]

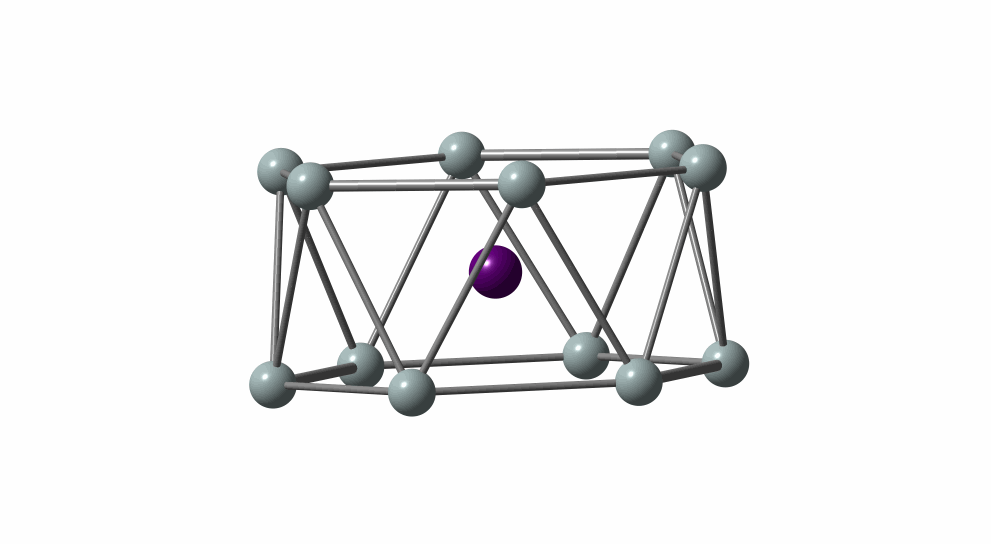

Supplement: Supplementary file 3 — jp1c10027_si_003.zip [file jp1c10027_si_003.zip › MnSi12+_gifs/295_e5.gif]

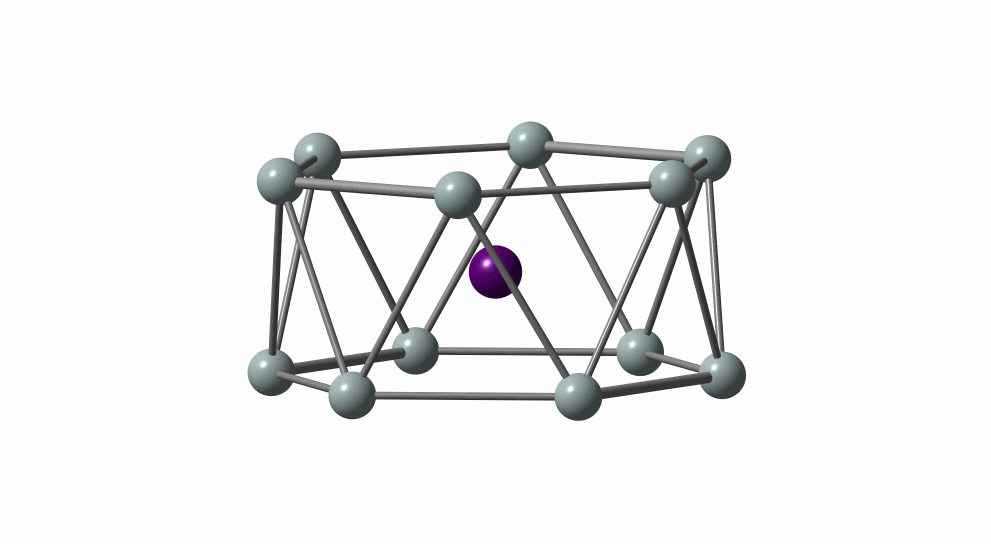

Supplement: Supplementary file 3 — jp1c10027_si_003.zip [file jp1c10027_si_003.zip › MnSi12+_gifs/300_a1.gif]

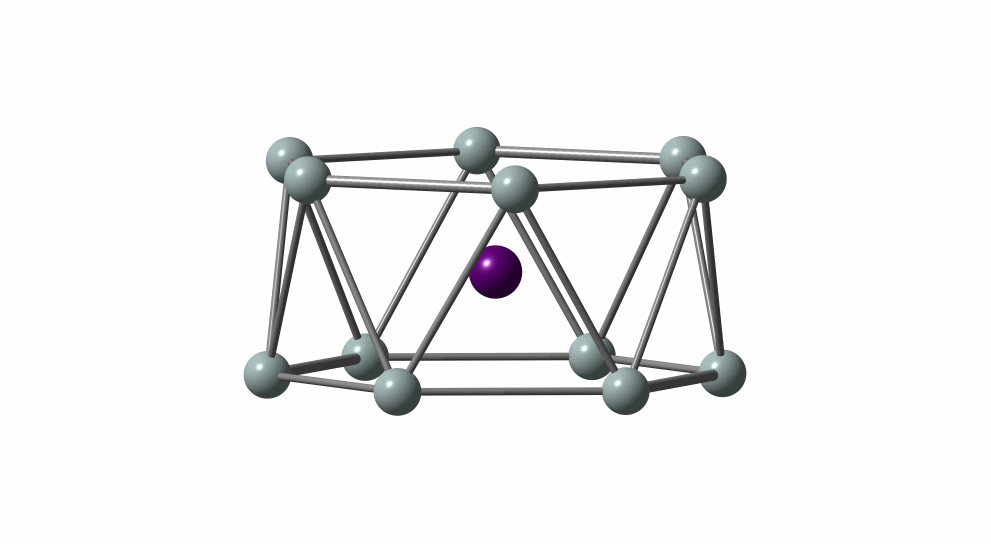

Supplement: Supplementary file 3 — jp1c10027_si_003.zip [file jp1c10027_si_003.zip › MnSi12+_gifs/314_b2.gif]

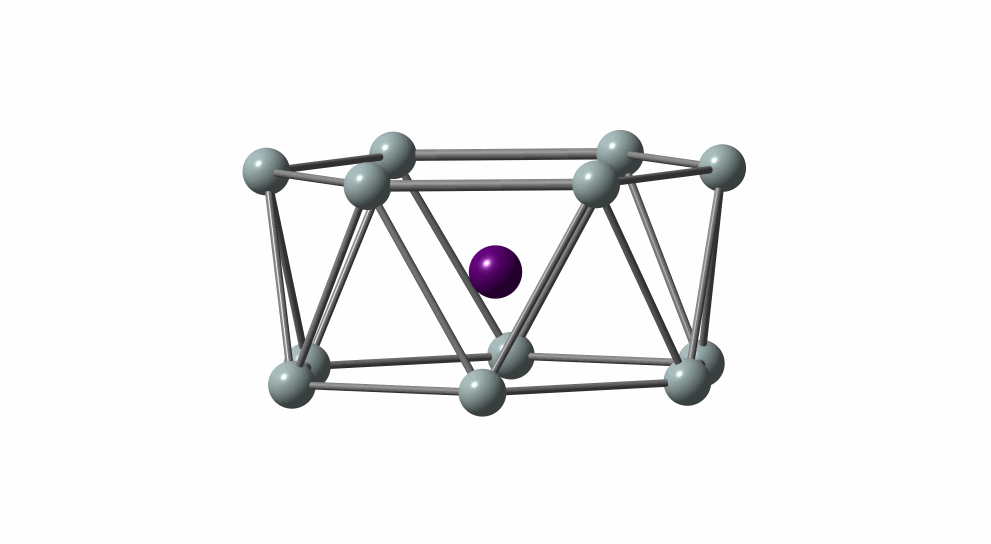

Supplement: Supplementary file 3 — jp1c10027_si_003.zip [file jp1c10027_si_003.zip › MnSi12+_gifs/364_e1.gif]

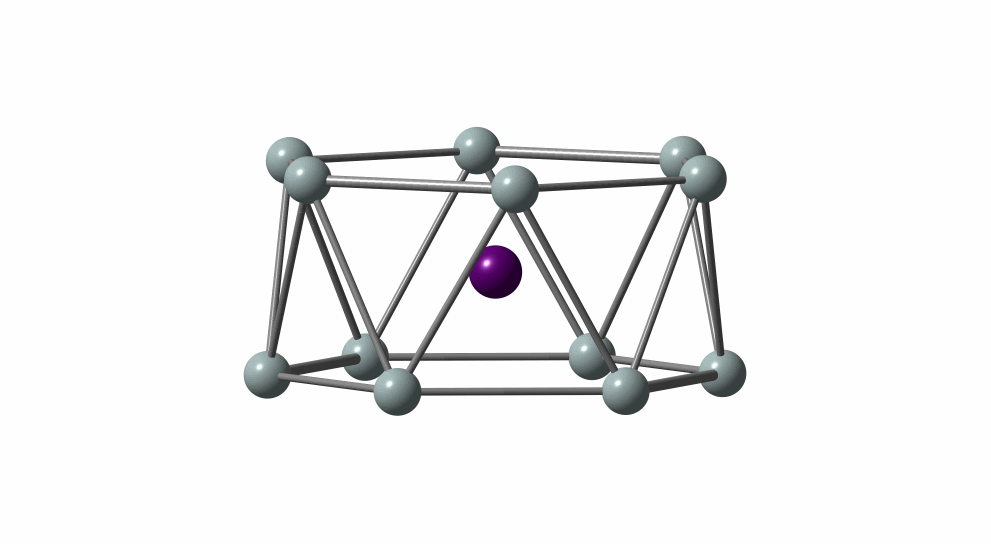

Supplement: Supplementary file 3 — jp1c10027_si_003.zip [file jp1c10027_si_003.zip › MnSi12+_gifs/386_a1.gif]
